# Spinal Cord Injury in Rats Disrupts the Circadian System

**DOI:** 10.1523/ENEURO.0328-18.2018

**Published:** 2018-12-21

**Authors:** Andrew D. Gaudet, Laura K. Fonken, Monica T. Ayala, Emily M. Bateman, Wolfgang E. Schleicher, Elana J. Smith, Heather M. D’Angelo, Steven F. Maier, Linda R. Watkins

**Affiliations:** 1 Department of Psychology and Neuroscience, University of Colorado Boulder, Boulder, Colorado 80301; 2 Center for Neuroscience, University of Colorado Boulder, Boulder, Colorado 80301; 3 Department of Psychology, College of Liberal Arts, University of Texas at Austin, Austin, Texas 78712; 4 Department of Neurology, Dell Medical School, University of Texas at Austin, Austin, Texas 78712; 5 Division of Pharmacology and Toxicology, University of Texas at Austin, Austin, Texas 78712

**Keywords:** biological clock, circadian rhythms, diurnal rhythms, neurotrauma, spinal cord injury

## Abstract

Spinal cord injury (SCI) perturbs many physiological systems. The circadian system helps maintain homeostasis throughout the body by synchronizing physiological and behavioral functions to predictable daily events. Whether disruption of these coordinated daily rhythms contributes to SCI-associated pathology remains understudied. Here, we hypothesized that SCI in rats would dysregulate several prominent circadian outputs including glucocorticoids, core temperature, activity, neuroinflammation, and circadian gene networks. Female and male Sprague Dawley rats were subjected to clinically relevant thoracic 9 moderate contusion SCI (or laminectomy sham surgery). Diurnal measures—including rhythms of plasma corticosterone (CORT), body temperature, and activity (using small implanted transmitters), and intraspinal circadian and inflammatory gene expression—were studied prior to and after surgery. SCI caused overall increases and disrupted rhythms of the major rodent glucocorticoid, CORT. Presurgery and sham rats displayed expected rhythms in body temperature and activity, whereas rats with SCI had blunted daily rhythms in body temperature and activity. In parallel, SCI disrupted intraspinal rhythms of circadian clock gene expression. Circadian clock genes can act as transcriptional regulators of inflammatory pathways. Indeed, SCI rats also showed dysregulated rhythms in inflammatory gene expression in both the epicenter and distal spinal cord. Our data show that moderate SCI in rats causes wide-ranging diurnal rhythm dysfunction, which is severe at acute time points and gradually recovers over time. Normalizing post-SCI diurnal rhythms could enhance the recovery of homeostasis and quality of life.

## Visual Abstract

**Figure:**
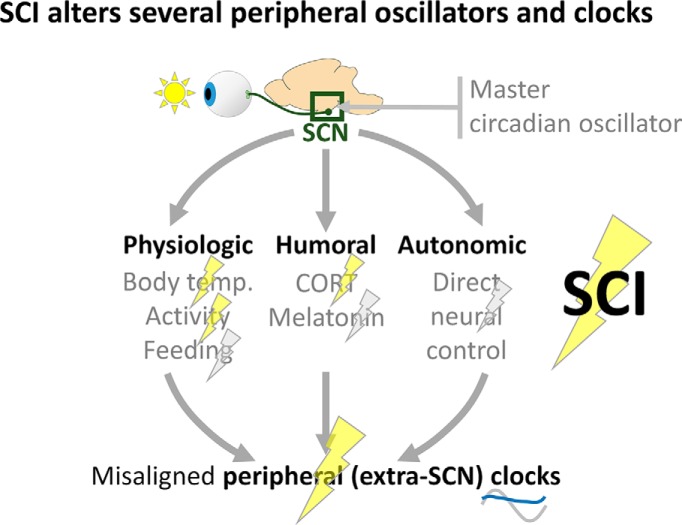

## Significance Statement

Spinal cord injury (SCI) can cause physiologic dysfunction throughout the body. Internal physiologic function is typically synchronized with the environment through the circadian system. Despite the crucial roles of the spinal cord and the circadian system in optimizing whole-body function, it remains unclear whether SCI alters diurnal rhythms. Here, we hypothesized that SCI would disrupt key circadian output and feedback mechanisms. Moderate SCI in rats caused widespread disruption of diurnal measures, including glucocorticoids, body temperature, locomotor activity, and intraspinal clock and inflammatory gene expression. We identify the circadian system as a novel potential target for SCI therapies.

## Introduction

Spinal cord injury (SCI) dysregulates a constellation of physiologic processes throughout the body. For instance, SCI suppresses peripheral immunity, which increases susceptibility to infection (Schwab et al., 2014). SCI predisposes to accumulating excess adipose tissue and to metabolic syndrome (Jones et al., 2003; Cragg et al., 2015; Sauerbeck et al., 2015). In addition, SCI causes sympathetic dysfunction, which contributes to cardiovascular issues such as autonomic dysreflexia and orthostatic hypotension (Alan et al., 2010; Inskip et al., 2012). Thus, SCI shifts prominent body systems away from a healthy equilibrium (i.e., physiologic homeostasis).

Physiologic homeostasis is regulated by the circadian system, which synchronizes organisms to daily fluctuations in their external environment by temporally organizing internal systems (Fonken and Nelson, 2014). Circadian rhythms are endogenous free-running rhythms (persisting even in constant darkness) lasting ∼24 h that are entrainable to external cues; when synchronized by light to day–night cycles, these rhythms are called “diurnal” rhythms. Initial circadian input occurs via light activation of retinal projections to the suprachiasmatic nucleus (SCN). SCN neurons entrain diurnal rhythms in other cells of the brain and body through neural (autonomic), humoral (e.g., glucocorticoids), and physiologic cues (external cues that coordinate biological rhythms; e.g., feeding, activity, and body temperature; Bedrosian et al., 2016). Circadian disruption can impair physiologic function, predispose to disease, and exacerbate postsurgery outcomes (Fonken et al., 2010; Li et al., 2011; Stevens et al., 2014). For example, disrupting the circadian clock with aberrantly timed light exposure following traumatic brain injury in rats increased neuronal death and cortical lesion volume, resulting in worsened sensorimotor and cognitive deficits (Li et al., 2016).

Research in rodents and humans suggests that SCI likely disrupts the circadian system. In mice, SCI transiently increased and disrupted daily expression rhythms of the major rodent glucocorticoid, corticosterone (CORT; Lucin et al., 2007). In rats, thoracic 3 (T3) spinal transection disrupted temperature rhythms for ∼14 d post-SCI (West et al., 2015). Together, these results imply that SCI may disrupt diurnal rhythms; however, these studies omitted sham surgery groups, which are essential given that surgery itself can disrupt these systems. Further, these studies did not explore other key circadian outputs (e.g., activity, circadian clock genes). In humans, cervical SCI disrupted diurnal body temperature rhythms at chronic times (Thijssen et al., 2011), and individuals with SCI often experience sleep disturbances (Giannoccaro et al., 2013); yet, diurnal rhythms in humans at acute-to-subacute post-SCI times remain understudied. Moreover, the existence and post-SCI regulation of a molecular circadian clock in spinal cord remains unstudied. Therefore, there is a need to understand the timing and extent of SCI-elicited circadian disruption, and how this could relate to the loss, and potential reacquisition, of physiologic homeostasis.

Here, we hypothesized that SCI in rats would disrupt physiologic, behavioral, and molecular diurnal rhythms. Female and male rats with thoracic SCI of moderate severity displayed a strong increase in, and arrhythmia of, a major circadian entraining factor, CORT. In addition, SCI dampened daily rhythms in body temperature and activity. Moreover, spinal cords from uninjured rats displayed robust diurnal regulation of clock genes; SCI dysregulated intraspinal expression of clock and inflammatory genes, both in epicenter and in distal lumbar spinal cord. We reveal that clinically relevant SCI in rats broadly disrupts circadian function, particularly acutely postinjury.

## Materials and Methods

### Surgery and animal care

These experiments were approved by University of Colorado Boulder Institutional Animal Care and Use Committee and conformed with ARRIVE (Animal Research: Reporting In Vivo Experiments) standards. In agreement with National Institutes of Health guidelines, male and female rats were included in all experiments. Rats had standard chow and filtered tap water available *ad libitum* and were maintained on a 12 h light/dark cycle. All surgeries occurred between zeitgeber time 2 (ZT2) and ZT11. Sprague Dawley sham/SCI rats (females, 200–250 × *g*; males, 320-380 × *g*; 2–3 months old; Envigo) received isoflurane anesthesia and a T8 laminectomy; SCI rats additionally received a moderate contusion injury (midline SCI; 150 kdyn, 1 s dwell; Infinite Horizon, Precision Systems and Instrumentation). Initial related studies involved pain testing, and inflammation was also studied, so analgesics were not given to any rats for consistency and for limiting confounds (Detloff et al., 2008; Cho et al., 2009; Ellis et al., 2016; Grace et al., 2016). All rats received prophylactic and postsurgery intraperitoneal antibiotics (gentamicin sulfate), subcutaneous saline for 5 d postinjury (dpi), and post-SCI bladder voiding twice daily (Gaudet et al., 2015, 2017).

### Tissue processing, histology, and analysis

To evaluate lesion size and spared tissue area, tissue was collected for immunohistochemistry (*n* = 7 male and female rats) at 7 dpi. Rats received an intraperitoneal pentobarbital overdose and were transcardially perfused with 0.9% saline, then 4% paraformaldehyde. Spinal cords were suspended in paraformaldehyde overnight, cyroprotected in 30% sucrose, and cryosectioned (16 μm; Gaudet et al., 2015). For immunohistochemistry, slides were incubated with 10% normal donkey serum (1 h), then with primary antibodies (overnight; mouse anti-glial fibrillary acidic protein (GFAP; 1:100; catalog #0869110, MP-Biomedicals) and rabbit anti-Iba1 (1:1000; catalog #019-19741, Wako Chemicals), then with secondary antibodies (2 h; Alexa Fluor-488 donkey anti-mouse (A-21202) and Alexa Fluor-546 donkey anti-rabbit (A-10040); both 1:500; Thermo Fisher Scientific) and DAPI (nuclear stain; catalog #D1306, Thermo Fisher Scientific). Images were captured on an Olympus IX81 Microscope and analyzed using Fiji (Schindelin et al., 2012).

### Locomotor testing

Locomotor recovery was assessed using the Basso, Beattie, and Bresnahan (BBB) scale (Basso et al., 1995; female sham: *n* = 6; SCI, *n* = 5; male sham: *n* = 7; SCI, *n* = 5) by two condition-blind observers before surgery; and at 1, 4, 7, 10, 14, 21, 28, 35, and 42 d postsurgery.

### Collection and measurement of plasma corticosterone

Rats were pair housed; cage mates had the same surgery (at start: female sham: *n* = 6; SCI, *n* = 10; male sham: *n* = 6; SCI, *n* = 10; two female and one male SCI rat died). Rats were acclimated to handling for 5 d; then, blood samples were collected from immobilized unanesthetized rats via tail nick (<500 μL/24 h). Blood was collected presurgery, and 2, 7, and 14 d postsurgery at ZT0, ZT6, ZT12, and ZT18 (starting at ZT0; CORT was typically the lowest). For dark-phase collections, dim red light headlamps were used (Fonken and Nelson, 2014). Samples were centrifuged (10,000 × *g*; 10 min) to isolate plasma. Plasma was used in a CORT ELISA (catalog #ADI-901-097, Enzo). Several samples (various rats and time points) had insufficient plasma, so were excluded, and samples that took longer than 3 min to collect were excluded (due to confounding stress-elicited CORT).

### Body temperature and locomotor activity measurement

Individually housed rats were implanted intraperitoneally with radiotelemetric transmitters (MiniMitter, Respironics; Fonken et al., 2012). After a 1 week recovery, prelaminectomy baseline activity/temperature was recorded for 1 week. Home cages on 12 TR-4000 receiver boards linked with DP-24 DataPorts (MiniMitter) continuously collected temperature/activity. Activity and body temperature were recorded for >6 weeks postsurgery.

Female and male experiments were completed separately due to a limited number of telemetry receivers. First, males [sham, *n* = 6; SCI, *n* = 5 (originally 6); one SCI rat transmitter did not transmit] were tested. Including shams was important: basic surgeries can disturb circadian rhythms (Farr et al., 1988). Sham/SCI rats received daily handling postsurgery (twice-daily bladder emptying or handling for shams; at approximately ZT2 and ZT10) and daily antibiotic/saline injections for 5 d postsurgery (approximately ZT2). Two weeks postsurgery, rats voided bladders independently, and daily checks continued without handling. After the male experiment, the experimental design was improved. For females (sham, *n* = 6; SCI, *n* = 6), presurgery handling (twice per day) acclimated the rats and reduced potential stress effects on postsurgery activity/temperature. Postsurgery antibiotic/saline injections (for 5 d postsurgery) occurred at afternoon (ZT10) animal care to limit the disruption of key early inactive phase rhythms.

### Diurnal regulation of gene expression

For the uninjured PCR study, tissues were collected from uninjured female/male rats across the day (ZT0, ZT6, ZT12, and ZT18; *n* = 6/sex/time point). Rats received an intraperitoneal pentobarbital overdose then were perfused with 0.9% saline. Tissues were flash frozen for PCR (L4–L5 was used for uninjured spinal cord analyses; T8 (lesion) and L4–L5 were used for postsurgery analyses). L4–L5 was used as a distal spinal cord site because it integrates hindpaw nociceptive information, which could provide useful information for future studies. L4–L5 was used for the uninjured analyses, since the T8 injury site was expected to have major shifts in gene expression (SCI vs sham, given that the lesion was present), whereas the lumbar spinal cord was predicted to have more minor SCI-elicited changes. For the sham/SCI PCR study, female/male rats received sham/SCI surgery (*n* = 6/sex, except for lumbar spinal cord, *n* = 4/sex; 2 d postsurgery collection). Before surgery, rats were handled to minimize postsurgery bladder care stress. SCI rats received postsurgery bladder care, and sham rats received similar handling (control for stress); the bladder care immediately before tissue collection was omitted to limit circadian disruption. Tissue from sham/SCI rats was collected at mid-light phase (ZT6; ∼48 h postsurgery) and at mid-dark phase (ZT18; ∼60 h postsurgery) at 2 d postsurgery. Tissue collections were completed within 1–2 h of the time point (e.g., ZT6; tissue collected at ZT5–ZT7). Subgroups of males and females were collected on the same days to minimize between-day differences and to enable sex comparisons.

Quantitative real-time PCR was completed as described previously (Fonken et al., 2018). Primers (Invitrogen) spanned exons (Table 1, for sequences). Gene expression was assessed in duplicate and is presented relative to β-actin. There were no significant differences in β-actin expression between groups, and male, female, sham, and SCI rat samples were all run on the same plates to ensure consistency. PCR results were analyzed using 2^-ΔΔCt^ and normalized with female sham-ZT6 set to 1.

**Table 1:** Primer sequences and related function of assessed RNAs

Gene	Protein function	Primer sequences (5´ to 3´)
*b-actin*	Housekeeping control	F: TTCCTTCCTGGGTATGGAAT R: GAGGAGCAATGATCTTGATC
*Bmal1*	Circadian clock gene, with *Clock*, activates *Per* and *Cry* transcription	F: AAAATGCAAGGGAGGCCCAC R: TCTAACTTCCGGGACATCGC
*Clock*	Circadian *Clock* gene, with *Bmal1*, activates *Per* and *Cry* transcription	F: CTGCTGACAAAAGCCAAGAT R: GACTTTCTTGAGCTTCTGGA
*Cry1*	Circadian *Clock* gene, with *Per*, represses *Bmal* and *Clock* transcription	F: GTGGTGGCGGAAACTGCTCTC R: ACTCTGTGCGTCCTCTTCCTGA
*Per1*	Circadian *Clock* gene, with *Cry*, represses *Bmal* and *Clock* transcription	F: GTGCAGGCTAACCAGGAATA R: GCGGAGAGTGTATTCAGATG
*Per2*	Circadian *Clock* gene, with *Cry*, represses *Bmal* and *Clock* transcription	F: ACAAGCGGCTGCAGTAGTGA R: TTCAAGGTTGCCAGCGTGCT
*Rev-erba (Nr1d1)*	Circadian *Clock* gene represses expression of core clock proteins	F: AGACGCTGTGCGTTTTGGAC R: TGTGGGAACTGAGAGAAGCC
*Cd68*	Inflammation expressed by microglia/macrophages; activation marker; cell homing and adhesion	F: CAAGCAGCACAGTGGACATTC R: CAAGAGAAGCATGGCCCGAA
*Iba1* *(AIF1)*	Inflammation expressed by all microglia/macrophages; increased with activation; binds calcium and actin	F: GGCAATGGAGATATCGATAT R: AGAATCATTCTCAAGATGGC
*IL-1b*	Proinflammatory cytokine; secreted mainly by microglia/macrophages	F: CCTTGTGCAAGTGTCTGAAG R: GGGCTTGGAAGCAATCCTTA
*IL-6*	Cytokine with mixed proinflammatory and anti-inflammatory roles	F: AGAAAAGAGTTGTGCAATGGCA R: GGCAAATTTCCTGGTTATATCC
*Mhc II*	Membrane-bound receptor; expressed by antigen-presenting cells (including microglia and macrophages); presents antigens	F: AGCACTGGGAGTTTGAAGAG R: AAGCCATCACCTCCTGGTAT
*Tnfa*	Proinflammatory cytokine; secreted mainly by microglia/macrophages	F: CAAGGAGGAGAAGTTCCCA R: TTGGTGGTTTGCTACGACG

F, Forward; R, reverse.

### Statistics

Data were analyzed (SigmaPlot 13.0, SYSTAT) using Student’s *t* test or nonparametric Mann–Whitney *U* test; or ANOVAs (one-, two-, or three-way ANOVA, as appropriate). Holm–Sidak *post hoc* tests were completed when appropriate for all tests involving more than two groups. Circadian data were analyzed using Circ Wave (https://www.euclock.org/results/item/circ-wave.html), which assesses independent circadian data (i.e., one individual contributes to a single data point) to identify waveforms and acrophases (rhythm peaks). Twice-daily postsurgery handling (voiding/injections) caused stress-elicited hyperthermia and hyperactivity in all groups and were excluded from Circ Wave analyses for clarity. Activity data were also analyzed using ClockLab 6.0 (Actimetrics); actograms and wavelets were assessed for each rat, and data from representative male sham and SCI individuals are presented. Actograms were scaled between 0 and 30. Researchers were blind to experimental group. Data were significant when *p* < 0.05. Data were plotted as the mean ± SEM.

## Results

### T8 spinal cord contusion (150 kdyn, 1 s dwell) causes tissue pathology and locomotor deficits

**Figure 1.**
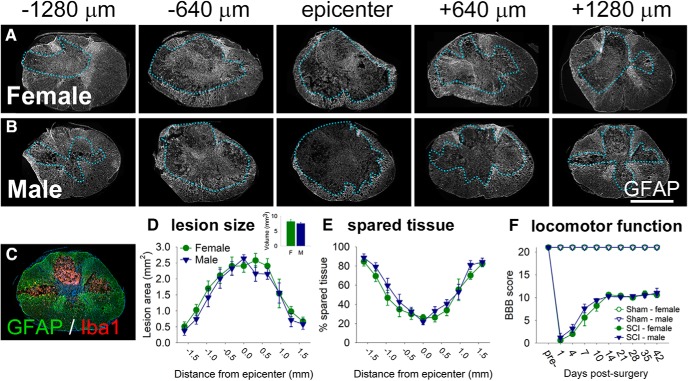
T9 contusion SCI (150 kdyn, 1 s dwell) causes extensive tissue loss at/near the epicenter with associated locomotor impairment. ***A***, ***B***, Spinal cords from female (***A***) and male (***B***) rats show substantial pathology and cavitation at 7 d post-SCI. GFAP immunoreactivity was used to visualize and assess tissue pathology; dotted lines outline the approximate lesion border in each section. ***C***, Example of GFAP (astrocytes, green) and Iba1 (microglia/macrophages, red) immunoreactivity in the 7 dpi lesion site (blue, nuclei; DAPI). GFAP^+^ astrocytes form the glial scar; Iba1^+^ macrophages/microglia exist in the epicenter and Iba1^+^ microglia are present in the lesion penumbra. ***D***, ***E***, Analysis of lesion area and volume (***D***) and the percentage spared tissue (***E***) at 7 d post-SCI. There were no significant sex differences in lesion size or the percentage of spared tissue. ***F***, Moderate T9 SCI caused substantial immediate locomotor deficits in female and male rats that recovered over time, as assessed in an open field using the BBB scale. There were no significant differences in BBB scores between female and male SCI rats. Scale bar, 1 mm.

First, we examined tissue damage in male and female rats at 7 dpi in this SCI model (Gaudet et al., 2017; Fig. 1). As expected, substantial tissue loss was observed at the T8 lesion epicenter (Fig. 1*D*); moving rostrocaudally away from the epicenter, there was progressively more tissue sparing (Fig. 1*E*). There were no significant differences in lesion size or tissue sparing in females versus males (lesion volume: females, 8.15 ± 0.70 mm^3^; males, 7.48 ± 0.46 mm^3^; *p* > 0.05).

Locomotor recovery after sham/SCI was assessed (Fig. 1*F*). At 1 dpi, average BBB scores indicated that SCI rats had movement of one hindlimb joint. By 42 dpi, SCI rats recovered frequent hindlimb stepping with no (or little) coordination (females: BBB score, 10.6 ± 0.6; males: BBB score, 11.0 ± 1.4).

### Spinal cord injury causes a transient increase and arrhythmia in plasma CORT

**Figure 2.**
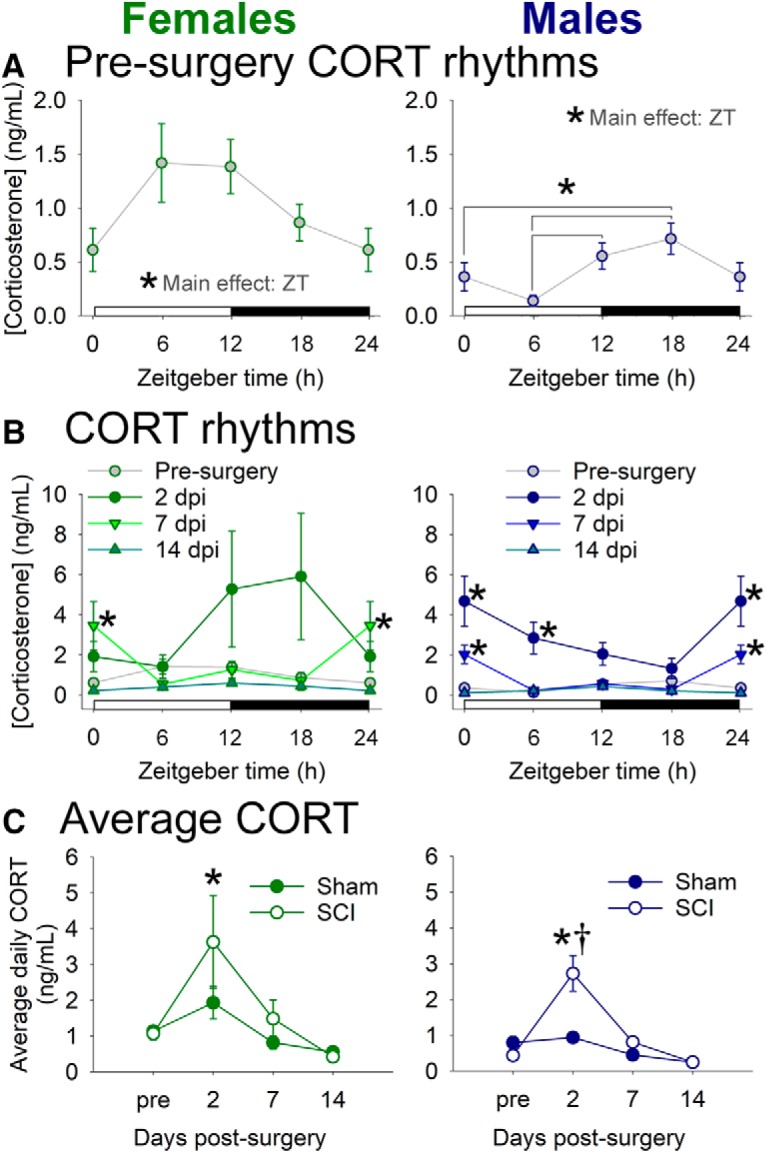
SCI in female and male rats disrupts diurnal rhythms in CORT. ***A***, Before surgery, female and male rats exhibited diurnal rhythms in CORT, with nadir levels at the beginning of the inactive (light) phase and peak levels toward the beginning of the active (dark) phase. ***B***, SCI increased CORT levels and altered CORT rhythms at 2 and 7 dpi; CORT rhythms were normalized by 14 dpi. ***C***, Average CORT level across the day in female and male SCI rats was increased at 2 dpi. *ZT differences or SCI vs presurgery, ANOVA with Holm–Sidak *post hoc* test, *p* < 0.05; †SCI vs sham at that time point, *p* < 0.05.

CORT release is regulated in a circadian manner; this steroid hormone maintains metabolic homeostasis across the day and helps to entrain extra-SCN circadian rhythms (Nicolaides et al., 2014). Here, we assessed whether SCI dysregulated plasma CORT levels and rhythms (Fig. 2). Before surgery, female and male rats showed peak trough patterns in plasma CORT (Fig. 2*A*; D’Agostino et al., 1982): CORT had cycle peak (acrophase) near early to mid-active phase (at approximately ZT12; females, ZT12: 1.4 ± 0.3 ng/ml; males, ZT12: 0.6 ± 0.1 ng/ml), and was lowest at the start of the inactive phase (ZT0; females: 0.6 ± 0.2 ng/ml; males: 0.4 ± 0.1 ng/ml). CORT levels were higher overall in presurgery females than males (Cavigelli et al., 2005). In females, CORT was unusally high at ZT6, which could have been related to a handling-elicited CORT increase (Gärtner et al., 1980). CORT was expressed rhythmically before injury in both females (*F*_(2,37)_ = 3.465, *p* < 0.05) and males (*F*_(2,33)_ = 6.647, *p* < 0.005).

SCI disrupted typical rhythms and significantly increased CORT levels at acute postinjury times (females: main effect of injury: *F*_(3,62)_ = 3.606, *p* < 0.05; males: main effect of injury: *F*_(3,70)_ = 22.584, *p* < 0.001). Females with SCI had increased CORT at 7 dpi (vs presurgery; at ZT0; *p* < 0.05); males with SCI showed significant CORT increases at 2 dpi (vs presurgery; at ZT0 and ZT6; *p* < 0.001) and 7 dpi (at ZT0; *p* < 0.05; Fig. 2*B*). For average CORT concentration across the day, SCI in female and male rats caused significant increases in average CORT at 2 d postsurgery (females: vs presurgery, *p* < 0.05; males: main effect of surgery; also at 2 dpi vs both presurgery and sham, *p* < 0.001; Fig. 2*C*).

### Spinal cord injury alters daily rhythms in body temperature

Body temperature and activity are two prominent outputs of the circadian system that also help to entrain circadian rhythms in cells throughout the body (Reebs and Mrosovsky, 1989; Buhr et al., 2010). To measure these parameters, rats were implanted with a small transmitter and were studied before sham/SCI, and from acute-to-chronic times postsurgery. (The studies on male and female rats were completed consecutively, due to the limited number of telemetry receivers; for details, see Materials and Methods.)

**Figure 3.**
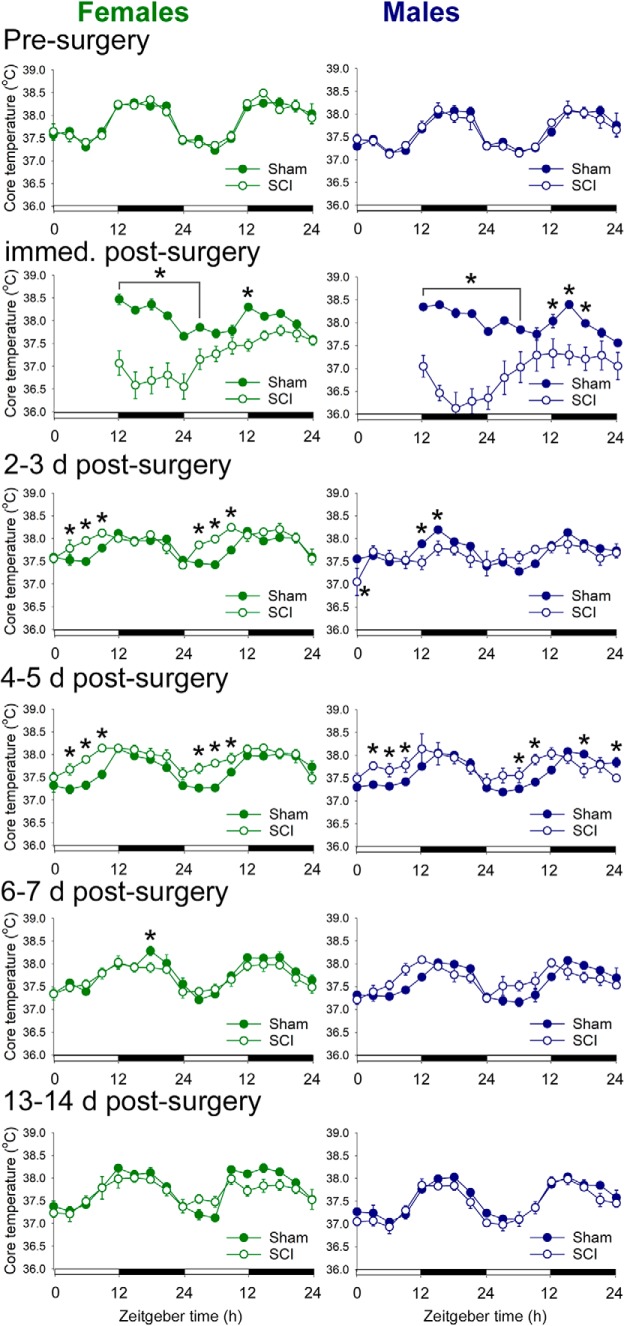
SCI in female and male rats disrupted diurnal rhythms of core body temperature. Before surgery, female and male rats exhibited typical diurnal core temperature rhythms. Immediately after surgery, SCI rats displayed hypothermia. SCI disrupted diurnal rhythms in core temperature between 2 and 5 d postsurgery. Diurnal temperature regulation was similar between sham and SCI rats by 13–14 d postsurgery. *p < 0.05 for sham vs SCI at that time point, ANOVA with Holm–Sidak *post hoc* test.

Before surgery, female and male rats displayed the expected diurnal rhythms in body temperature: core temperature was higher during the active phase, and lower during the inactive phase (female-SCI presurgery acrophase, ZT16.6; male-SCI presurgery acrophase, ZT17.3; Fig. 3). Immediately after surgery (i.e., after replacing postsurgery rats on transmitter boards), SCI but not sham rats experienced hypothermia (lasted ∼18 h; ANOVA main effect: female: *F*_(1,155)_ = 31.664, *p* < 0.001; male: *F*_(1,142)_ = 24.663, *p* < 0.001; and significant differences at specific times; Fig. 3).

After initial post-SCI hypothermia, female rats showed significant SCI-elicited temperature dysregulation. By 2 d postsurgery, female sham and SCI rats both showed approximately typical rhythms in core temperature (Circ Wave; all showed rhythmicity). Compared with sham rats, female SCI rats had shifted (advanced) acrophases between 2 and 6 d postsurgery and increased inactive phase temperatures between 2 and 5 d postsurgery (2–6 d sham acrophases: ZT15.5, ZT15.7, ZT14.9, and ZT15.9, respectively; 2–6 dpi SCI acrophases: ZT12.0, ZT12.9, ZT13.0, and ZT14.5, respectively; ANOVA of main effect of treatment and injury–time interaction, 2–3 d postsurgery: *F*_(1,203)_ = 6.678, *p* < 0.05; sham vs SCI, 2 d postsurgery: ZT3, ZT6, and ZT9; 3 d postsurgery: ZT3, ZT6, and ZT9, *p* < 0.05; 4–5 d postsurgery: *F*_(1,203)_ = 12.207, *p* < 0.01; sham vs SCI: 6 d postsurgery: ZT18, *p* < 0.05; inactive phase temperature: SCI > sham, 15 d postsurgery, *p* < 0.05). SCI rats displayed no significant differences versus sham at 7, 13, or 14 d postsurgery (7 dpi: ANOVA, no main effect of treatment, *F*_(1,203)_ = 0.982; 13–14 dpi: ANOVA, no main effect of treatment, *F*_(1,203)_ = 1.147, *p* > 0.05).

Male rats showed a similar pattern to females of SCI-elicited temperature dysregulation, but with somewhat delayed recovery of rhythms. Sham rats at 2–3 dpi showed approximately typical rhythms in core temperature (2 and 3 d postsurgery sham acrophases: ZT13.3 and ZT16.2; all showed rhythmicity); conversely, male SCI rats at 2–3 dpi did not have significant core temperature rhythms (ANOVA main effect of treatment: *F*_(1,186)_ = 0.688, *p* > 0.05). Male SCI rats displayed shifted acrophases and increased inactive phase temperatures compared with sham rats between 4 and 5 d postsurgery (acrophases: sham, 4 d postsurgery: 16.4; SCI, 4 dpi: 13.7; sham, 5 d postsurgery: 16.4; SCI, 5 dpi: 12.5; ANOVA main effect of treatment: *F*_(1,186)_ = 0.657, *p* > 0.05; inactive phase temperature: SCI > sham, 4–5 d postsurgery, *p* < 0.05). There were no significant differences caused by SCI in core temperature at 6–7 d postsurgery (ANOVA, no main effect of treatment: *F*_(1,186)_ = 0.400, *p* > 0.05) nor at 13–14 d postsurgery (ANOVA, no main effect of treatment: *F*_(1,186)_ = 2.794, *p* > 0.05).

**Figure 4.**
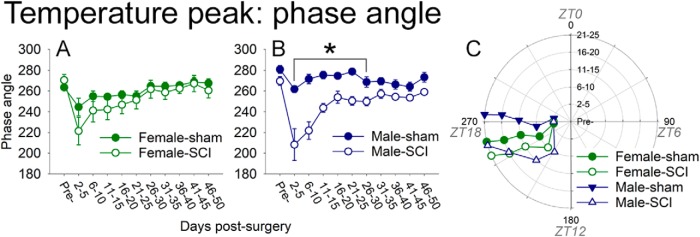
SCI significantly advanced temperature acrophase in males, but not females. Temperature phase angles (in bins of 4–5 d) were studied in female and male rats before surgery, then up to 50 d postsurgery. ***A***, Compared with sham rats, females with SCI showed no significant difference in phase angle after surgery. ***B***, SCI males had significantly advanced temperature acrophases from 2–5 d through 26–30 d postsurgery. ***C***, Phase plot showing how SCI affected the phase angle over time postsurgery (up to 25 d for clarity). Angle indicates the time of acrophase; radial distance depicts time postsurgery. *p < 0.05.

To understand more broadly whether SCI influenced temperature rhythms, daily temperature was processed in ClockLab in grouped multiday “bins” (Fig. 4). Before surgery, the peak daily temperature phase occurred in females at an angle of 263.5^°^ (or ZT17.8) and in males at 275.2^°^ (or ZT18.3). After surgery, females with SCI showed no significant difference in phase from shams (*p* > 0.05, two-way repeated-measures ANOVA with Holm–Sidak *post hoc* test). In contrast, males with SCI displayed significantly advanced temperature phases: SCI males had earlier temperature acrophase starting at 2–5 d postsurgery [ANOVA main effect of treatment: *F*_1,81_ = 43.299, *p* < 0.001; 2–5 d postsurgery: male sham rats, phase angle 261.9^°^ ± 3.4^°^ (ZT17.5); male SCI rats, phase angle 208.3 ± 15.3^°^ (ZT13.9); *p* < 0.05], and these SCI advanced rhythms continued through all 5 d bins examined through 26–30 d postsurgery [26–30 d postsurgery: male sham rats, phase angle 268.7 ± 4.9^°^ (ZT17.9); male SCI rats, phase angle 249.9 ± 4.1^°^ (ZT16.7); *p* < 0.05]. At 31–35 d postsurgery and beyond, male SCI rat temperature acrophases were not significantly different from those of male sham rats [e.g., 31–35 d postsurgery: male sham rats, 269.5 ± 3.5^°^ (ZT18.0); male SCI rats, 257.2 ± 4.^o^ (ZT17.1); *p* > 0.05].

Overall, SCI altered the average core temperature at early postinjury times—particularly due to increased temperature during the inactive phase—and showed substantial recovery at subacute times. SCI males (vs females) displayed significant and longer-lasting shifts in temperature acrophase.

### Spinal cord injury disrupts amount and diurnal rhythms of activity

Activity patterns are a strong index of the integrity of the circadian system. Although SCI clearly reduces activity, it is also possible that it disrupts activity rhythms. Rhythmic activity is important for entraining peripheral circadian rhythms (Reebs and Mrosovsky, 1989; Tahara et al., 2017). Before surgery, rats showed typical diurnal patterns of activity, with more activity during the active phase (female SCI rats presurgery acrophase, ZT15.1; male SCI rats presurgery acrophase, ZT17.9; counts in active phase: females, 65 ± 2%; males, 67 ± 2%).

**Figure 5.**
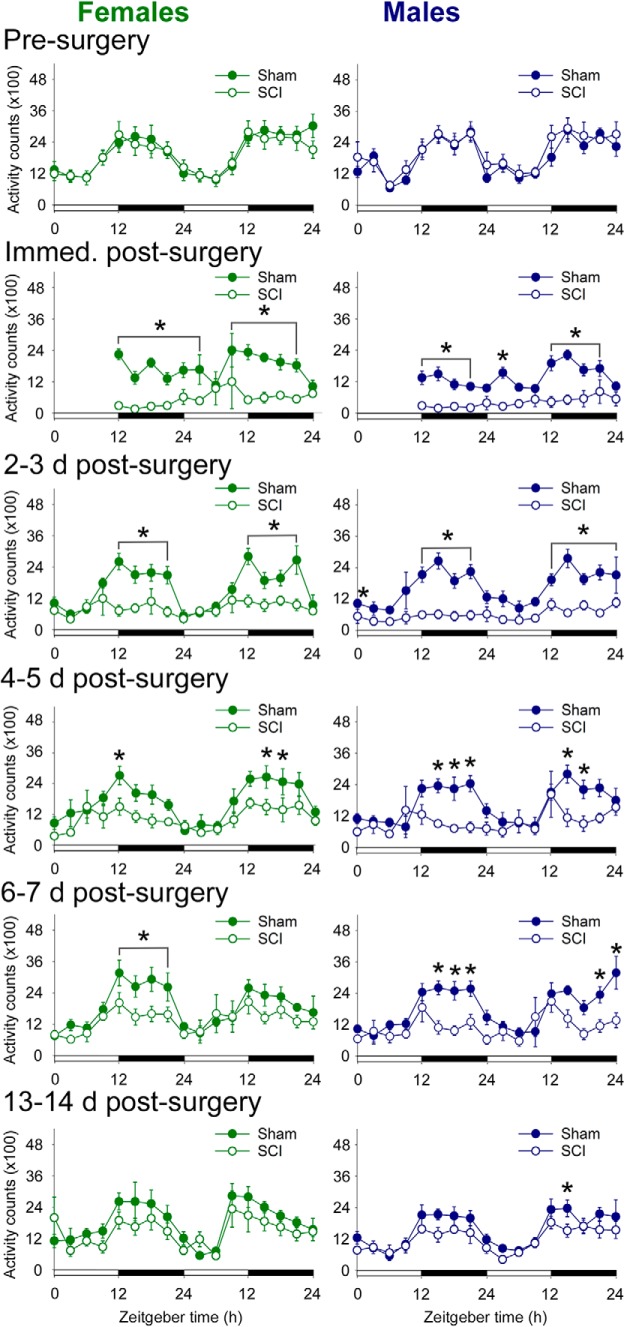
SCI in female and male rats disrupted diurnal rhythms in activity. Data show cumulative activity counts per 3 h time period. Before surgery, female and male rats exhibited typical diurnal rhythms in activity, with rats more active in the dark phase. As expected, SCI rats were less active at acute times postinjury (up to 7 d postsurgery). Interestingly, at subacute (14 d postsurgery) to chronic times, SCI rats regained activity levels and diurnal rhythms in activity that were similar to those of sham rats. *p < 0.05 for sham vs SCI at that time point, ANOVA with Holm–Sidak *post hoc* test.

After surgery, differences in activity between sham and SCI rats occurred mainly during the active phase (Fig. 5). Sham rats showed relatively typical activity levels and diurnal activity rhythms by 2–3 d postsurgery (2 and 3 d postsurgery sham acrophases: female, ZT15.2 and ZT16.2; male, ZT15.8 and ZT16.8). In contrast, female and male SCI rats had strongly reduced activity throughout the day at 2 dpi (total counts: female sham rats, 13,000 ± 2000; female SCI rats, 7000 ± 2000; *p* < 0.05; male sham rats, 13,200 ± 800; male SCI rats, 3200 ± 800; *p* < 0.001). Although this pattern of post-SCI reduced activity continued for days, there was some recovery of diurnal activity rhythms over the first 5 dpi. At 6–7 dpi, female and male rats with SCI showed further recovery of activity, but not 6 dpi, although their overall activity remained significantly lower than that of sham rats. Patterns of activity at 6 d postsurgery and beyond were not significantly different between sham and SCI rats in both females and males (percentage counts in active phase at 6 d postsurgery: female sham rats, 70 ± 2%; female SCI rats, 63 ± 3%; *p* > 0.05; male sham rats, 70 ± 2%; male SCI rats, 62 ± 3%; *p* > 0.05).

At 13–14 d postsurgery, female and male SCI rats had activity patterns that were more similar to shams (acrophases: female sham rats, 13.9; female SCI rats, 13.3; male sham rats, 16.2; male SCI rats, 15.8; total counts in active phase at 14 dpi: female sham rats, 9000 ± 900; female SCI rats, 7000 ± 1000; *p* > 0.05; male sham rats, 8500 ± 500; male SCI rats, 6600 ± 500; *p* < 0.05). These patterns were even more similar at a chronic post-SCI time point (acrophases: female sham rats, 15.8; female SCI rats, 15.7; male sham rats, 16.1; male SCI rats, 15.5). Therefore, SCI transiently dampens activity levels, and the timing of post-SCI recovery of daily activity counts and rhythms parallels the recovery of other circadian measures.

### Spinal cord injury delays recovery of 24 h activity rhythms

**Figure 6.**
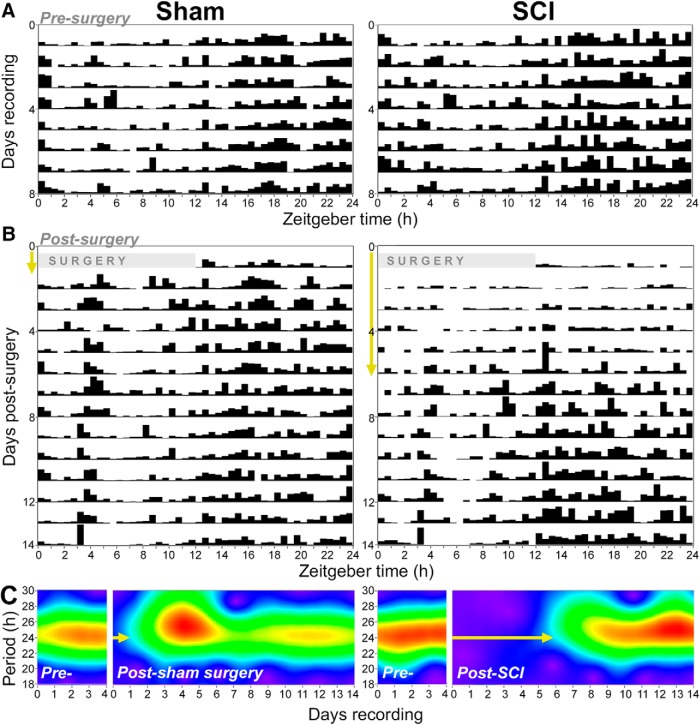
Rats with SCI show delayed the recovery of diurnal activity rhythms. These representative data are from one male sham and one male SCI rat. ***A***, ***B***, Actograms display continuous measures of activity across the day (ZT; *x*-axis) and over time postsurgery (*y*-axis). ***A***, Before surgery, these rats in the sham and SCI groups had expected diurnal patterns of activity (i.e., increased activity between ZT12 and ZT24; higher black bars). ***B***, After surgery, sham rats quickly recover more typical activity rhythms, whereas SCI rats show delayed postsurgery recovery of rhythms. Yellow arrows highlight approximate latency to the recovery of 24 h rhythms; also shown in ***C***. ***C***, Wavelet analysis shows that before surgery rats display strong 24 h rhythms, and that sham and SCI rats have different postsurgery latencies to recover 24 h activity rhythms (yellow arrows). The sham rat recovered 24 h rhythms within ∼1 d postsurgery, whereas the SCI rat recovered 24 h rhythms at ∼6 d postsurgery. The intensity of rhythm across days is represented by the color continuum: purple (minimal rhythm) through blue and green to red (intense rhythm).

To establish postsurgery latency to recovery of 24 h activity rhythms, actogram and wavelet analyses were performed. These qualitative assessments help visualize diurnal rhythms. Data from one male sham rat and one male SCI rat (both representative) are presented. Before surgery, the rats displayed expected rhythms in activity (Fig. 6*A*). After sham surgery, rats recovered relatively typical rhythms within ∼1 d postsurgery. In contrast, after SCI, rats recovered 24 h activity rhythms approximately 6–7 d postsurgery (Fig. 6*B*,*C*, yellow arrows). Therefore, SCI substantially delayed the recovery of diurnal activity rhythms.

### Spinal cord injury disrupts clock gene expression in epicenter and lumbar spinal cord

**Figure 7.**
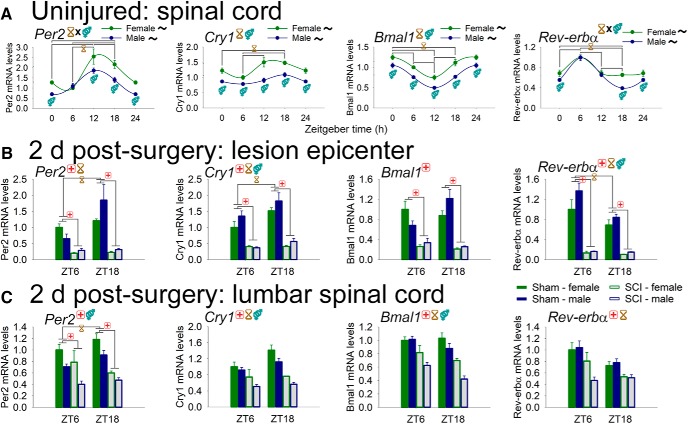
Diurnal regulation of clock genes in spinal cord from uninjured rats and from sham/SCI rats at 2 d postsurgery. ***A***, Female and male rat spinal cords express clock genes, and clock gene expression varies through the day. *Per2*, *Cry1*, *Bmal1*, and *Rev-erba* displayed rhythmic expression in the spinal cord. Females generally expressed higher levels of expression of clock genes. ***B***, SCI disrupts clock gene expression at the lesion epicenter. Clock gene expression at ZT6 and ZT18 was assessed. Sham spinal cords showed clock gene variation between the two time points (e.g., *Per2*, *Cry1*, and *Rev-erba*). After SCI, injury epicenters showed strongly reduced expression and ablated diurnal variation of these four clock genes, suggesting circadian disruption. ***C***, SCI disrupts clock gene expression in spinal cord distal to injury. The lumbar spinal cord, which was not directly injured by contusion, showed decreased expression of the four clock genes examined in SCI vs sham rats. There were also time-of-day differences for all genes presented, and a significant main effect of sex for *Per2*, *Cry1*, and *Bmal1*. Black ∼ indicates that females or males for that gene show significant rhythm; red **+** indicates injury difference (sham vs SCI), *p* < 0.05; yellow hourglass indicates time difference, *p* < 0.05; blue gender symbol indicates sex difference, *p* < 0.05. Symbols at the top of each graph indicate the significant main effect; symbols above/below data indicate significant interactions.

Rhythms of circadian “clock” gene expression exist throughout the body; however, clock gene expression has not been systematically characterized in the spinal cord. Thus, first we sought to establish how clock genes are regulated by time of day in the uninjured rat spinal cord (Fig. 7*A*). Our data reveal that clock genes are expressed rhythmically in the L4–L5 spinal cord. The “core clock” components *Per2*, *Cry1*, and *Bmal1* were all regulated in spinal cord by time and sex [*Cry1*, *Bmal1*: main effect of time (*p* < 0.001) and sex (females > males, *p* < 0.001); *Per2*: significant time–sex interaction, *F*_3,44_ = 2.953, *p* < 0.05] and were expressed rhythmically across the day (all in both females and males; acrophases: *Per2*: female, ZT15.2 ± 1.2; male, ZT12.2 ± 1.2; *Cry1*: female, ZT16.3 ± 1.4; male, ZT16.8 ± 1.5; *Bmal1*: female, ZT23.0 ± 1.4; male, ZT0.8 ± 1.3). Similarly, *Rev-erbα*, a transcription factor in a secondary circadian feedback loop, was regulated in spinal cord by time of day (interaction with sex: *F*_(3,44)_ = 3.953, *p* < 0.05) and was rhythmically expressed (in females and males; *Rev-erbα* acrophases: female, ZT6.0 ± 1.4; male, ZT6.6 ± 1.1).

To establish whether SCI alters molecular circadian rhythms, rats were subjected to T8 sham (laminectomy) or SCI surgery, and tissue was collected at 2 d postsurgery in the light (ZT6) or dark (ZT18) phase. Clock gene expression was assessed in T8 epicenter and lumbar spinal cord (Fig. 7*B*,*C*). In T8 epicenter, SCI (vs sham surgery) substantially downregulated key clock genes *Per2*, *Cry1*, *Bmal1*, and *Rev-erbα* (*F*_(1,45)_ = 45.7, 113.2, 82.1, and 135.6, respectively; all *p* < 0.001; main effect at ZT6 and ZT18; Fig. 7*B*). In addition, there were time-of-day differences in sham rats that were abolished by SCI in several genes (*Per2*, *Cry1*, and *Rev-erbα*; *post hoc* test), and there were sex differences in *Cry1* and *Rev-erbα* expression (main effect; males higher).

Dysregulation of circadian genes by SCI extended beyond the lesion site. Lumbar spinal cord from SCI (vs sham) rats displayed significantly reduced expression of *Per2*, *Cry1*, *Bmal1*, and *Rev-erbα* (*F*_(1,30)_ = 40.7, 48.4, 62.2, and 23.8, respectively; all *p* < 0.001, all main effects; SCI also downregulated *Per2* at ZT6 and ZT18; Fig. 7*C*). In addition, there were main effects on clock gene expression of time (*Cry1*, *Bmal1*, and *Rev-erbα*) and sex (*Per2*, *Cry1*, and *Bmal1*). Additional clock genes dysregulated by SCI included *Per1* and *Clock* (Table 2).

**Table 2:** : Expression of additional clock (*Per1*, *Clock*) and inflammatory (*Iba1*, *Mhc II*) genes at 2 d postsurgery in the lesion epicenter and lumbar spinal cord (data only for genes not shown in figures)

	Female				Male				Significant differences	
	ZT6		ZT18		ZT6		ZT18			
Gene	Sham	SCI	Sham	SCI	Sham	SCI	Sham	SCI	Main effect	Interactions
*Per1*										
Lesion Lumbar	1.00 ± 0.10 1.00 ± 0.05	0.39 ± 0.06 1.04 ± 0.29	1.19 ± 0.07 0.97 ± 0.09	0.23 ± 0.03 0.70 ± 0.03	1.01 ± 0.17 0.78 ± 0.08	0.35 ± 0.06 0.57 ± 0.07	2.01 ± 0.57 0.84 ± 0.12	0.34 ± 0.01 0.50 ± 0.02	SCI: ZT6 + 18 SCI, sex	Sham × ZT N.S.
*Clock*										
Lesion Lumbar	1.00 ± 0.16 1.00 ± 0.03	0.32 ± 0.03 0.71 ± 0.10	1.21 ± 0.18 1.07 ± 0.06	0.27 ± 0.04 0.64 ± 0.04	0.90 ± 0.18 0.94 ± 0.07	0.36 ± 0.09 0.53 ± 0.07	1.56 ± 0.33 0.83 ± 0.08	0.29 ± 0.03 0.45 ± 0.04	SCI SCI	Sham × ZT Sham × ZT
*Iba1*										
Lesion Lumbar	1.00 ± 0.21 1.00 ± 0.07	0.75 ± 0.03 1.36 ± 0.04	0.86 ± 0.08 0.96 ± 0.08	0.93 ± 0.09 1.38 ± 0.13	0.69 ± 0.09 1.32 ± 0.05	0.41 ± 0.07 2.06 ± 0.45	0.59 ± 0.04 1.23 ± 0.12	0.78 ± 0.14 1.54 ± 0.18	Sex N.S.	SCI × ZT;ZT6 × inj. N.S.
*Mhc II*										
Lesion Lumbar	1.00 ± 0.17 1.00 ± 0.19	0.21 ± 0.05 0.50 ± 0.05	0.82 ± 0.09 0.54 ± 0.21	0.21 ± 0.03 0.54 ± 0.18	0.65 ± 0.14 1.59 ± 0.38	0.23 ± 0.05 0.86 ± 0.14	0.79 ± 0.14 1.09 ± 0.30	0.22 ± 0.05 0.54 ± 0.11	SCI SCI, sex	N.S. N.S.

For statistical comparisons (far right column): SCI, significant main effect of SCI vs sham; ZT, significant main effect of time (ZT6 vs ZT18); sex, significant main effect of sex; Sham × ZT, significant interaction between sham and ZT; N.S., no significant difference.

### Spinal cord injury disrupts inflammatory gene expression in epicenter and lumbar spinal cord

**Figure 8.**
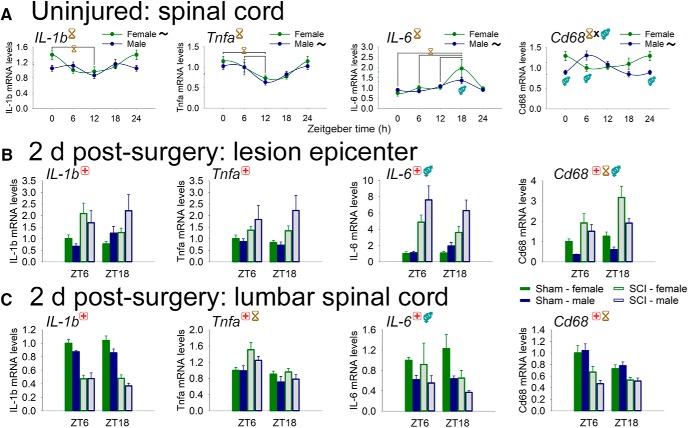
Diurnal regulation of inflammatory genes in spinal cord from uninjured rats, and from sham/SCI rats at 2 d postsurgery. ***A***, Female and male rat spinal cord expression of inflammatory genes varies through the day. *IL-1b*, *Tnfa*, and *Cd68* displayed time-of-day expression differences in the spinal cord. Rhythmic expression was observed in *IL-1b* (females), *Tnfa* (males), *IL-6* (males), and *Cd68* (males). Sex affected intraspinal *IL-6* and *Cd68* gene expression. ***B***, SCI alters inflammatory gene expression at the lesion epicenter. SCI increased expression of *IL-1b*, *Tnfa*, *IL-6*, and *Cd68* expression. Epicentre *IL-6* was also regulated by sex, and *Cd68* was also regulated by ZT and by sex. ***C***, SCI modulates inflammatory gene expression in spinal cord distal to injury. SCI reduced lumbar spinal cord expression of *IL1b*, *IL-6*, and *Cd68*, and increased the expression of *Tnfa*. There were also significant main effects of ZT (*Tnfa*, *Cd68*; both lower at ZT18) and sex (*IL-6*; females higher than males). Black ∼ indicates that females or males for that gene show significant rhythm; red **+** indicates injury difference (sham vs SCI), *p* < 0.05; yellow hourglass indicates time difference, *p* < 0.05; blue gender symbol indicates sex difference, *p* < 0.05. Symbols at the top of each graph indicates significant main effect; symbols above/below data indicate significant interactions.

Inflammatory genes in the CNS can also be regulated by time of day, and this likely confers differential immunocompetence across the day (Fonken et al., 2015). In the healthy adult rat spinal cord, several inflammatory genes were expressed differentially across the day (Fig. 8*A*): the proinflammatory cytokines *IL-1b* (main effect of time: *F*_(3,43)_ = 3.54, *p* < 0.05; females had significant daily rhythm, *p* < 0.05) and *Tnfa* (main effect of time: *F*_(3,44)_ = 5.57, *p* < 0.005; males had significant daily rhythm, *p* < 0.01); the inflammatory cytokine *IL-6* (main effect of time: *F*_(1,44)_ = 6.99, *p* < 0.001; males had significant daily rhythm, *p* < 0.005); and the microglial/macrophage activation marker *Cd68* (significant interaction of time and sex: *F*_(3,44)_ = 5.51, *p* < 0.005; males had significant daily rhythm, *p* < 0.005).

SCI significantly dysregulated inflammatory gene expression in epicenter and lumbar spinal cord (main effects; Fig. 8*B*,*C*). *IL-1b* was significantly upregulated in SCI (vs sham) rats in the T8 lesion epicenter but was downregulated in the lumbar spinal cord (*F*_(1,45)_ = 11.9 and *F*_(1,30)_ = 144.7, respectively; both *p* < 0.001; main effects). *Tnfa* was upregulated by SCI in both the epicenter and the lumbar spinal cord (*F*_(1,45)_ = 12.9 and *F*_(1,29)_ = 7.9, respectively; both *p* < 0.001; main effects; also main effect of time: lumbar). *IL-6* expression was increased in SCI rats in the epicenter, but was reduced in the lumbar spinal cord (*F*_(1,44)_ = 43.6, *p* < 0.001; *F*_(1,29)_ = 4.5, *p* < 0.05, respectively; main effects; also main effect of sex in both tissues). *Cd68* expression was increased by SCI in the epicenter, but was downregulated in the lumbar spinal cord [*F*_(1,44)_ = 32.6 and *F*_(1,29)_ = 31.2, respectively; both *p* < 0.001; main effects; also main effect of time (epicenter and lumbar) and sex (epicenter); SCI also altered intraspinal expression of *Iba1*, an RNA expressed by microglia/macrophages, and *Mhc II*, an antigen-presenting molecule expressed by microglia/macrophages; Table 2]. Thus, SCI robustly dysregulated intraspinal clock and inflammatory gene expression.

## Discussion

**Figure 9.**
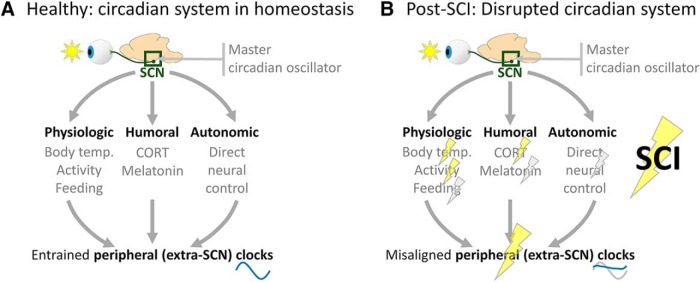
SCI disrupts diurnal rhythms. ***A***, Diurnal rhythm control under homeostatic conditions. Initial circadian input occurs via light activating specialized retinal ganglion cells that project directly to the SCN. The SCN is the master circadian oscillator; in turn, this regulates extra-SCN (“peripheral”) rhythms via direct and indirect routes. The SCN controls peripheral clocks directly via autonomic control (sympathetic and parasympathetic innervation), whereas the SCN controls peripheral clocks indirectly through the regulation of physiologic and humoral factors. Appropriately entrained clocks throughout the body (likely every cell) optimize organismal performance for time of day. ***B***, Diurnal rhythm control is disrupted by SCI. Our data suggest that SCI disrupts rhythms of key zeitgeber times, including body temperature, activity, and CORT level (yellow bolts), which could disrupt peripheral clock entrainment. In addition, data from other groups suggest that SCI also disrupts additional entraining factors (e.g., feeding, melatonin, and autonomic input; gray bolts). Ultimately, prolonged SCI-elicited disruption of these entraining factors could contribute to the loss of homeostasis and suboptimal repair.

This study used female and male rats to determine whether SCI dysregulated acute-to-chronic physiologic and behavioral rhythms and related molecular outputs. Moderate T8 spinal cord contusion dysregulated plasma CORT—a key humoral output of and feedback signal for the circadian system—particularly at 2 and 7 d postsurgery. In addition, SCI dampened diurnal rhythms in locomotor activity and body temperature. SCI strongly decreased the expression of several clock genes in the epicenter and lumbar spinal cord, and also altered the expression of intraspinal inflammatory genes at two times of day. Therefore, moderate SCI in a rat model severely disrupts daily rhythms in CORT, activity, body temperature, and intraspinal gene expression. The physiologic parameters studied gradually reacquired more typical daily rhythms over time. Our results suggest that moderate contusion SCI causes widespread, transient disruption of physiologic homeostasis (Fig. 9).

### Locomotor recovery after SCI

As expected, rats with SCI showed a substantial deficit in hindlimb movement at 1 dpi, and the rats recovered frequent stepping by the chronic 42 dpi time point. Locomotor recovery had not previously been assessed in this model (a T8 150 kdyn contusion with 1 s dwell; Putatunda et al., 2014; Gaudet et al., 2017; Hook et al., 2017). Interestingly, the post-SCI locomotor recovery presented here complements the findings of others: our average BBB score of 10.6 in female 42 dpi rats supports that this injury severity is between the severities reported previously (vs 150 and 200 kdyn with 0 s dwell; Scheff et al., 2003).

### SCI disrupts diurnal rhythms and physiologic homeostasis

Diurnal rhythms help to optimize organism function across the day; thus, disrupting diurnal rhythms likely shifts the physiology of an animal away from homeostasis. Prolonged diurnal disruptions have detrimental effects for the individual. Here, we identify SCI as a disruptor of several prominent physiologic rhythms: CORT, core temperature, and activity.

Glucocorticoids are steroid hormones that help to synchronize extra-SCN circadian rhythms (Balsalobre et al., 2000). This is possible because SCN-derived signals entrain CORT rhythms to light and SCN cells lack CORT receptors, whereas most non-SCN cells express CORT receptors. In this manner, CORT can modulate peripheral circadian clocks directly (e.g., CORT–receptor complex drives *Per1* and reduces *Rev-erbα* transcription; Oster et al., 2006; Dickmeis, 2009; Surjit et al., 2011). CORT is potently upregulated by immune activation and helps to resolve inflammatory responses; however, CORT is also critically involved in stress responses (Frank et al., 2013) and basic homeostatic functions (Nicolaides et al., 2014). Given these roles of CORT in health and pathology, we hypothesized that SCI would dysregulate CORT rhythms. Indeed, male and female rats with acute SCI had increased CORT levels and disrupted CORT rhythms. Previous work supports our findings: female mice with T9 contusion SCI had acute, transient dysregulation of glucocorticoid rhythms (Lucin et al., 2007), although no sham mice were included. A recent study by Pruss et al. (2017) found that SCI in mice increased serum CORT levels (T1 SCI > T9 SCI; 3 dpi CORT with no time of day specified); similarly, humans within 96 h post-SCI showed increased serum cortisol levels. SCI in mice and humans substantially increases susceptibility to peripheral infection (pneumonia). In mice, SCI-elicited CORT increases could be quenched by removing the major source of systemic CORT, the adrenal glands, but adrenalectomy did not reduce the frequency of pneumonia (Pruss et al., 2017). Interestingly, transplanting denervated adrenal glands into adrenalectomized SCI mice normalized CORT levels and limited susceptibility to pneumonia (Pruss et al., 2017; but also see Oster et al., 2006). Thus, accelerating the recovery of CORT rhythms could help to regain homeostasis, including diurnal rhythms.

We also revealed that daily rhythms in body temperature were significantly altered at acute post-SCI times and gradually recovered. Immediate post-SCI hypothermia lasted ∼24 h (hypothermia not observed in shams). Body temperature acrophase was particularly disrupted in male rats with SCI, which had altered temperature acrophase that persisted through 30 dpi. Others also observed SCI-elicited temperature arrhythmia in rats (West et al., 2015). Similarly, humans with acute trauma (including SCI) commonly experience hypothermia, which can copresent with other pathologies and lead to death (Kirkpatrick et al., 1999). In humans with chronic SCI, diurnal regulation of core body temperature was disrupted after cervical, but not thoracic, SCI (although acute post-SCI rhythms were not assessed; Thijssen et al., 2011). Body temperature rhythms help coordinate peripheral circadian rhythms (Buhr et al., 2010); thus, optimizing post-SCI recovery of body temperature rhythms could accelerate the recovery of homeostasis.

Activity rhythms were transiently dampened by SCI, which could have more widespread implications for the circadian system. For example, timed activity can be used to help strengthen diurnal rhythms: in mice with a mutation in core circadian clock machinery, scheduled (late night) wheel running strengthened molecular and physiologic rhythms (Schroeder et al., 2012). Behavioral activity feeds back to the SCN: exercise in mice suppresses SCN neuron activity, and exercising at the proper time of the cycle increases SCN diurnal rhythm amplitude (van Oosterhout et al., 2012). In humans, exercising at abnormal times (during the night) phase shifts diurnal rhythms (Youngstedt et al., 2016). Therefore, post-SCI diurnal rhythms may be strengthened by optimizing the time of day of key activities, including rehabilitation, social activity, and feeding.

Glucocorticoids, body temperature, and activity modulate circadian oscillators throughout the body, so SCI-elicited dysregulation could alter daily rhythms more broadly. Indeed, typical diurnal rhythms in body function can be disrupted in humans by SCI (e.g., sleep; Giannoccaro et al., 2013) and by related medical interventions (Li et al., 2011), so expediting the recovery of diurnal rhythms after SCI could improve neuroprotection and functional outcomes.

It is important to note that we had limited telemetry receivers, so we completed the body temperature and activity transmitter study separately in males and females. In addition, limitations were noted after completing the male study and methods were improved for the female portion (e.g., rats were acclimated to twice-daily handling before surgery), which could have affected the consistency of the results. Despite this, the results were remarkably similar between males and females, both for sham and SCI rats, and differences existed between sham and SCI groups. Our experience and data also highlight that typical postsurgery rodent care and behavioral testing in many research laboratories (e.g., twice-daily rodent care during the light/inactive phase) likely further disrupts daily rhythms (as well as sleep), and could be a confound to the factor under study (McEwen, 2006). Future SCI studies could improve their design by having the daily injections and major care immediately before the active phase, and by having a second potential daily care time during the dark phase or at another minimally disruptive time (e.g., using shifted light schedules in animal housing, if helpful). In addition, given that intense rodent care during the beginning of the inactive phase is likely a strong disruptor of rhythms, future studies could compare whether this type of disruption affects anatomic and neurologic outcomes compared with a “circadian-optimized” care schedule. These findings would have translational relevance, since early post-SCI patients in hospitals likely experience excess circadian disruptions (that could be improved with relatively simple interventions).

Another distinction between studies described herein is that the telemetry study was completed with individually housed rats (to enable measuring the temperature and activity of a single rat), whereas all other studies were completed with rats housed in pairs. This is important to note, given that housing rats individually is a stressor (Von Frijtag et al., 2000; Westenbroek et al., 2005) and can impair recovery after CNS injury (Craft et al., 2005; Karelina et al., 2009; Gaudier-Diaz et al., 2017). Furthermore, single housing may reduce relative activity (vs having a cage mate) and therefore likely worsens other aspects of recovery (e.g., locomotor recovery; Van Meeteren et al., 2003).

### SCI alters intraspinal expression and diurnal regulation of clock and inflammatory genes

At 2 d postsurgery, SCI remarkably reduced the expression of clock genes observed in the lesion epicenter, and a more modest (but significant) decrease also occurred in the lumbar spinal cord. In the lesion epicenter, this is likely affected by altered cell composition: sham T8 spinal cords contain typical CNS cellular constituents, whereas SCI T8 epicenters include various activated immune, glial, and other cells (Gaudet and Fonken, 2018). Epicenter-localized cells could have different clock gene levels that cause the massive decrease observed. Another possibility is that SCI dysregulates a finely tuned circadian and inflammatory gene expression network: immune cell-specific circadian gene dysregulation can provoke inflammatory responses (Nguyen et al., 2013; Fonken et al., 2016; Segal et al., 2018); conversely, the induction of cytokines such as TNF-α and IL-1β can suppress the expression and/or function of circadian genes (Cavadini et al., 2007). Indeed, intraspinal *Tnfa* and *IL-1b* diurnal regulation inversely correlated with *Per2* expression, both in uninjured spinal cord and in the SCI epicenter. Similarly, Rev-erbα reduces IL-6 expression (Gibbs et al., 2012); here, diurnal variation in *Rev-erbα* and *IL-6* levels were inversely related in uninjured spinal cord, and the SCI-elicited decrease in epicenter *Rev-erbα* paralleled an increase in *IL-6*. These findings underscore the potential relevance of clock and inflammatory gene cross talk. In addition, SCI downregulated clock genes even distal to lesion in lumbar spinal cord, where cell infiltration likely has limited influence. Overall, decreased and arrhythmic clock gene expression by SCI could broadly regulate immune and homeostatic function in the CNS.

Inflammatory gene expression was differentially affected by SCI in epicenter and lumbar spinal cord. In the epicenter, most genes assessed were upregulated; conversely, most genes assessed in the lumbar spinal cord were downregulated. L4–L5 spinal cord was chosen as a distal spinal cord site because it integrates hindpaw nociceptive information. At later times post-SCI, excess below-level intraspinal inflammation likely contributes to SCI-elicited neuropathic pain (Hains and Waxman, 2006; Detloff et al., 2008), and we found that this contusion model causes neuropathic pain symptoms by 14 dpi in female and male rats (Gaudet et al., 2017). The reduced expression of inflammatory markers at 2 dpi may represent an initial remote intraspinal dampening of the immune response, which may precede chronic neuroinflammation that exacerbates neuropathic pain and pathology.

Here, two time points [middle of the light (ZT6) and dark (ZT18) phase] were used to assess clock and inflammatory gene expression at 2 d post-SCI. This is a limitation of the study, since with two time points it is not possible to make broader conclusions about the effects of SCI on circadian phase, amplitude, and average expression. Future studies could examine the expression at additional times of day. Further, the disruption of circadian outputs persisted beyond 2 dpi, including rhythms of serum CORT, body temperature, and activity (all were disrupted until at least 7 dpi), yet our study only assessed gene expression changes at 2 d postsurgery. Gene expression was assessed at 2 d postsurgery because robust changes in the circadian system were seen at this time, and because early changes in lesion pathology and inflammation likely have strong effects on recovery trajectory (Noble et al., 2002; Kigerl et al., 2009; Gaudet et al., 2018; McCreedy et al., 2018). It is also important, however, to understand how clock and inflammatory gene patterns shift through subacute and chronic times postsurgery. In the future, studying circadian gene expression changes at additional times post-SCI would provide more information about the robustness and duration of the effects of SCI on the circadian system.

### Future directions

Our study highlights that SCI disrupts whole-body functions, yet unresolved questions remain about post-SCI circadian disruption and related potential therapies. As shown in Figure 9, SCI alters the regulation of several circadian outputs. Daily rhythms are entrained by light input to the retina, influencing the SCN, which is likely protected from changes caused by SCI [e.g., unlike most cells of the body, SCN neurons lack CORT receptors; Balsalobre et al., 2000; Woodruff et al., 2016). However, peripheral rhythms are synchronized and entrained by other circadian oscillators, including CORT, body temperature, activity, and autonomic circuits (Buijs et al., 2016), that are perturbed by SCI. Therefore, the disruption of circadian outputs could contribute to more widespread circadian disruption throughout the body. Here, a moderate T8 spinal cord contusion was used; future studies could establish whether more severe injuries or injuries at other spinal levels (e.g., cervical or high thoracic, which would more strongly perturb autonomic function; Inskip et al., 2009) differentially alter postinjury circadian dynamics. In addition, our study did not address whether circadian disruption was simply a result of injury, or whether disruption itself further exacerbated outcomes. Future studies could determine whether amplifying post-SCI rhythms using, for example, timed exercise, feeding, light/dark schedule manipulations, and injections of circadian-related drugs, could improve recovery measures (discussed more below). The strengthening of post-SCI rhythms could also influence other long-term recovery measures, such as survival, neuroprotection, chronic pain, autonomic function, and general health. Further, since altering circadian rhythms affects other aspects of metabolism, metabolic cage telemetry and calorimetry (Gaudet et al., 2016) could reveal whether boosting post-SCI rhythms improves short-term and long-term metabolic health. Finally, to better understand the effect of SCI on circadian rhythms, SCI researchers could adopt other models and strategies frequently used in circadian research, such as the *PER::luciferase* transgenic rodent, which uses the clock gene promoter from *Per* to express the bioluminescent *luciferase* gene and enables studying rhythm synchrony/dysregulation *in vitro* (Yamazaki et al., 2000) and *in vivo* (Tahara et al., 2012).

If SCI-elicited circadian disruption worsens recovery and metabolism, amplifying diurnal rhythms using entraining strategies soon after injury could expedite recovery (Roenneberg and Merrow, 2016). For instance, improving the darkness of rooms during nights (e.g., minimize brightness of lights/monitors; use red lights and filters) and incorporating bright morning light (e.g., having an outdoor-facing window in the room; Engwall et al., 2014), feeding during the day (Stokkan et al., 2001; Fonken et al., 2010), and optimizing the time of day for rehabilitation/activity (Schroeder et al., 2012) could improve sleep–wake cycles and re-entrain diurnal rhythms. Using these and other strategies to accelerate post-SCI recovery of homeostasis could boost key early recovery processes and overall postinjury outcomes.

### Conclusions

In conclusion, we used a clinically relevant rat spinal contusion model to assess how SCI affects circadian dynamics. We established that moderate thoracic SCI has broad effects on diurnal rhythms, including disrupted rhythms of plasma CORT levels, activity, body temperature, and intraspinal gene expression. SCI caused robust diurnal rhythm disruption at acute post-SCI times (2 and 7 dpi; across various circadian measures); SCI rats recovered more typical diurnal rhythmicity by subacute-to-chronic times (14–42 dpi). SCI-associated disruption of these key regulators of physiologic homeostasis may feed back to impede SCI recovery. Future discovery and clinical SCI studies could incorporate measures of circadian function, which may reveal post-SCI “chronotherapies” that help to regain homeostasis and improve recovery.

## Synthesis

Reviewing Editor: Robert Kalb, Northwestern University Decisions are customarily a result of the Reviewing Editor and the peer reviewers coming together and discussing their recommendations until a consensus is reached. When revisions are invited, a fact-based synthesis statement explaining their decision and outlining what is needed to prepare a revision will be listed below. The following reviewer(s) agreed to reveal their identity: NONE. Note: If this manuscript was transferred from JNeurosci and a decision was made to accept the manuscript without peer review, a brief statement to this effect will instead be what is listed below.

Both reviewers were positively inclined towards this work. An important limitation is “... one major limitation is that gene expression data in the lesioned and sham operated controls were examined just at two time points across the day, in contrast to the data acquired in untreated controls. This allows to generally detect (most) changes, but does not inform whether phases, amplitude and basal expression levels are altered....”. IN addition there are issues with the statistics. The authors must address all the concerns raised by the reviewers that require rewriting or re-analysis of data. If there is more gene expression data that the authors are withholding they are strongly encouraged to introduce it into the ms.

## Author Response

Synthesis of Reviews:

Computational Neuroscience Model Code Accessibility Comments for Author: There is cloud data that can not be accessed. Please correct:

Link to software (CircWave) does not appear to work when entered into an internet browser. The first link I do find upon searching for CircWave states that “CircWave analyses independent circadian data (ie, one individual contributes one data point). Typically such data sets are produced in circadian brain research where techniques such as micro array chips, in situ hybridisations, immunocytochemistry, northern, western, and soutern blots, etc...are used.” I do not believe that was the case here, i.e. no repeated data points in a given animal. An updated link would remedy confusion over how cosinor analysis was performed.

RESPONSE: We have updated the link to the software. In further considering effective use of the software, we agree that it is inappropriate to use the program for repeated data points. As such, we have removed inappropriate analyses and related discussion of rhythmicity (e.g., for glucocorticoids, temperature, and activity figures). CircWave is appropriate for analyzing the gene expression data presented in Fig. 6 and 7. We updated the description of circadian analysis using CircWave to be more specific:

Page 10: Circadian data were analyzed using CircWave (https://www.euclock.org/results/item/circ-wave.html), which assesses independent circadian data (i.e., one individual contributes to a single data point) to identify waveforms and acrophases (rhythm peaks).

Furthermore, to better understand how SCI altered circadian rhythms, we performed additional analysis on the post-SCI temperature data and have added a new figure (Figure 4). The data show that males, in particular, have SCI-elicited disruptions in temperature rhythms: SCI males (but not females) show significantly advanced acrophases of temperature rhythms. Discussion of this is included in the Results:

Page 14-15: “To understand more broadly whether SCI influenced temperature rhythms, daily temperature was processed in ClockLab in grouped multi-day ”bins“ (Fig. 4). Prior to surgery, peak daily temperature phase occurred in females at an angle of 263.5o (or ZT17.8) and in males at 275.2o (or ZT18.3). After surgery, females with SCI showed no significant difference in phase from shams (p &gt; 0.05; two-way RM ANOVA with Holm-Sidak post-hoc). In contrast, males with SCI displayed significantly advanced temperature phases: SCI males had earlier temperature acrophase starting at 2-5 d post-surgery (ANOVA main effect of treatment: F1,81=43.299, p &lt; 0.001) (2-5 d post-surgery: male-sham, phase angle 261.9o {plus minus} 3.4o (=ZT17.5); male-SCI, 208.3o {plus minus} 15.3o (=ZT13.9), p &lt; 0.05) and these SCI-advanced rhythms continued through all 5 d bins examined through 26-30 d post-surgery (26-30 d post-surgery: male-sham, 268.7o {plus minus} 4.9o (=ZT17.9); male-SCI, 249.9o {plus minus} 4.1o (=ZT16.7), p &lt; 0.05). At 31-35 d post-surgery and beyond, male SCI rat temperature acrophases were not significantly different from male sham rats (e.g., 31-35 d post-surgery: male-sham, 269.5o {plus minus} 3.5o (=ZT18.0); male-SCI, 257.2o {plus minus} 4.o (=ZT17.1), p &gt; 0.05).”

Significance Statement Comments for Author: Both authors found the work an important step towards understanding the relationship to circadian rhythms and SCI

Synthesis Statement for Author: Both reviewers were positively inclined towards this work. An important limitation is “... one major limitation is that gene expression data in the lesioned and sham operated controls were examined just at two time points across the day, in contrast to the data acquired in untreated controls. This allows to generally detect (most) changes, but does not inform whether phases, amplitude and basal expression levels are altered....”. In addition there are issues with the statistics. The authors must address all the concerns raised by the reviewers that require rewriting or re-analysis of data. If there is more gene expression data that the authors are withholding they are strongly encouraged to introduce it into the ms.

RESPONSE: We thank the reviewers for their positive comments, and for improving the quality of the manuscript with their thorough and constructive feedback.

We agree that only including two post-surgery timepoints is a limitation of the study; however, as mentioned by the reviewers, completing these studies with an additional two timepoints would have required substantially more rats. Further, grant funding that supported this work has expired, so we do not have the support to complete additional related studies. We did not withhold gene expression data related to this manuscript. We addressed these concerns by more carefully discussing the results throughout the manuscript (for instance, stating that “expression was altered” rather than “rhythms were ablated”), and by adding further clarification of the strengths and limitations of our study in the discussion.

In addition, we agree that there was some ambiguity regarding the statistics used in the manuscript, and we have addressed these concerns by removing statistical analyses that were inappropriate based on type of data and analysis, and by clarifying the statistical methods used.

Please see responses to reviewers below for more details regarding these improvements.

Reviewer #1

The manuscript is clearly written and includes informative, well-organized figures. The authors designed their experiments well by including sham operated controls, in contrast to earlier studies addressing similar questions. Thereby, the paper provides a sound descriptive baseline upon which future studies can rely. However, one major limitation is that gene expression data in the lesioned and sham operated controls were examined just at two time points across the day, in contrast to the data acquired in untreated controls. This allows to generally detect (most) changes, but does not inform whether phases, amplitude and basal expression levels are altered. While I understand that taking more samples across the day-night cycle would have meant a substantial increase in animal numbers and logistic efforts, this choice still limits interpretation of the data. For example, it is not possible to state that rhythms are “ablated” (Page 20) based on two time points. One can only conclude that expression at these time points is altered compared to the sham or untreated controls.

RESPONSE: We thank the reviewer for their positive comments. We address comments about rhythms in the first response below.

Comments to the Authors: In their manuscript „Spinal cord injury in rats disrupts diurnal rhythms“, the authors report perturbation of circadian physiology and gene expression upon moderate to severe spinal cord contusion in rats. The authors observe altered plasma corticosterone, body temperature and locomotor activity rhythms as well as changed patterns of gene expression for circadian clock and inflammatory genes in the spinal cord itself, both at the lesion site and at a site more distant from the lesion.

The manuscript is clearly written and includes informative, well-organized figures. The authors designed their experiments well by including sham operated controls, in contrast to earlier studies addressing similar questions. Thereby, the paper provides a sound descriptive baseline upon which future studies can rely. However, one major limitation is that gene expression data in the lesioned and sham operated controls were examined just at two time points across the day, in contrast to the data acquired in untreated controls. This allows to generally detect (most) changes, but does not inform whether phases, amplitude and basal expression levels are altered. While I understand that taking more samples across the day-night cycle would have meant a substantial increase in animal numbers and logistic efforts, this choice still limits interpretation of the data. For example, it is not possible to state that rhythms are ”ablated” (Page 20) based on two time points. One can only conclude that expression at these time points is altered compared to the sham or untreated controls.

RESPONSE: Regarding the gene expression data, we agree that the collection of only two timepoints over 24 hours after spinal cord injury is a limitation of the study. As mentioned, the number of timepoints was limited because of the number of animals required and due to the logistics of performing the SCIs and post-SCI care at manageable intervals. We agree that the wording with regards to the “rhythms” should be changed to more accurately reflect the results in the manuscript.

Accordingly, we removed the description of “ablated rhythms” in the first paragraph of the Discussion to more accurately reflect the results. In addition, we added a brief paragraph to the discussion to address this limitation.

Page 26-27: “Here, two timepoints (middle of the light (ZT6) and dark (ZT18) phase) were used to assess clock and inflammatory gene expression at 2 d post-SCI. This is a limitation of the study, since with two timepoints it is not possible to make more broad conclusions about the effects of SCI on circadian phase, amplitude, and average expression. Future studies could examine expression at additional times of day. Further, disruption of circadian outputs persisted beyond 2 dpi, including rhythms of serum CORT, body temperature, and activity (all were disrupted until at least 7 dpi) - yet our study only assessed gene expression changes at 2 d post-surgery. Gene expression was assessed at 2 d post-surgery because robust changes in the circadian system were seen at this time; however, it is also important to understand how clock and inflammatory gene patterns change into sub-acute and chronic times post-surgery. In the future, studying circadian gene expression changes at additional times post-SCI would provide more information about the robustness and duration of SCI's effects on the circadian system.”

Other points:

1) Did rats receive analgetics post operation? Could one use analgetic treatment to discriminate between pain effects on the circadian system and other, perhaps more directly inflammation or lesion related effects?

RESPONSE: Our studies were initially completed in parallel with testing for neuropathic pain, although no rats in this study were subjected to pain-related testing. In addition, buprenorphine can affect inflammation (e.g., Cho et al., 2009 PMID: 19409968; Hemshekhar et al., 2017 PMID: 28572711) (which was studied as well), so to avoid potential confounds we did not provide buprenorphine. Rats were monitored closely and recovered well. This is now clarified in the Materials and Methods:

Page 6: “Initial related studies involved pain testing, and inflammation was also studied, so analgesics were not given to any rats for consistency and limiting confounds (Detloff et al., 2008; Cho et al., 2009; Ellis et al., 2016; Grace et al., 2016).”

2) Page 8: Discuss potential effects of the experimental design improvement between male and female experiments also in the main body of the text?

RESPONSE: We agree that this is an important point, that was also mentioned by Reviewer #2. We clarified this detail in the Results:

Page 12: “(The studies on male and female rats were completed consecutively, due to limited number of telemetry receivers - see Materials and Methods for details.)”

In addition, we added a paragraph to the Discussion:

Page 24-25: “It is important to note that we had limited telemetry receivers, so we completed the body temperature and activity transmitter study separately in males and females. In addition, limitations were noted after completing the male study and methods were improved for the female portion (e.g., rats were acclimated to twice daily handling prior to injury), which could have affected the consistency of the results. Despite this, the results were remarkably similar between males and females - both for sham and SCI rats - and differences existed between sham and SCI groups. Our experience and data also highlight that typical post-surgery rodent care and behavioral testing in many research labs (e.g., twice-daily rodent care during the light/inactive phase) likely further disrupts daily rhythms (as well as sleep), and could be a confound to the factor under study (e.g., (McEwen, 2006). Future SCI studies could improve design by having the daily injections and major care immediately before the active phase, and by having a second potential daily care time during the dark or at another minimally disruptive time (using shifted light schedules in animal housing, if helpful). In addition, given that intense rodent care during the beginning of the inactive phase is likely a strong disruptor of rhythms, future studies could compare whether this type of disruption affects anatomical and neurologic outcomes compared to a ”circadian-optimized“ care schedule. These findings would have translational relevance, since early post-SCI patients in hospitals likely experience excess circadian disruptions (that could be improved with relatively simple interventions).”

3) Figure 1: add arrows to indicate lesion area? Difficult to see for the non-expert...

RESPONSE: Thank you for pointing out this issue. We added a dotted line in each panel of Fig. 1A highlighting the extent of the lesion and added appropriate text to the figure legend.

4) Figure 2: consider changing the scale to better visualize the flatter CORT rhythms in pre-surgery animals?

RESPONSE: We added panels (Fig. 2A) to better visualize pre-surgery CORT levels. In carefully considering these data, it is noted that there is an unusually high CORT value for females at ZT6. This is stated and discussed briefly in the Results:

Page 11-12: CORT levels were higher overall in pre-surgery females than males (Cavigelli et al., 2005). In females, CORT was unusally high at ZT6, which could have been related to a handling-elicited CORT increase (Gartner et al., 1980).

5) Page 21: Rev-erba contains a negative GRE element, arguing rather for direct regulation by glucocorticoids (see [1] and [2])

RESPONSE: We thank the reviewer for highlighting this error and for the addition of relevant citations. We have now corrected this in the Discussion:

Page 21: “In this manner, CORT can modulate peripheral circadian clocks directly (e.g., CORT-receptor complex drives Per1 and reduces Rev-erbα transcription) (Oster et al., 2006; Dickmeis, 2009; Surjit et al., 2011).”

6) Page 22: Evidence that transplanted adrenal glands show circadian production of glucocorticoids can be found in [3]. Was a similar protocol used in the cited study by Pruss et al.?

RESPONSE: The citation mentioned is an interesting example of the role of the adrenal glands in circadian regulation. In looking through the methods in the Pruss paper, it appears the adrenal transplant is similar/identical between the papers, though the Oster paper is not cited in Pruss et al. We altered the text (Page 22) to acknowledge/cite the Oster study and point the reader in that direction for further information.

References

1. Torra, I.P., et al., Circadian and glucocorticoid regulation of Rev-erbalpha expression in liver. Endocrinology, 2000. 141(10): p. 3799-806.

2. Surjit, M., et al., Widespread negative response elements mediate direct repression by agonist-liganded glucocorticoid receptor. Cell, 2011. 145(2): p. 224-41.

3. Oster, H., et al., The circadian rhythm of glucocorticoids is regulated by a gating mechanism residing in the adrenal cortical clock. Cell Metab, 2006. 4(2): p. 163-73.

Reviewer #2

This manuscript advances the field of SCI-induced systemic dysregulation by providing a wide-ranging view of changes in behavioral, physiological, and molecular rhythms occurring after SCI. The study leaves avenues for future research to better assess interrelationships and mechanisms between these dysfunctions on the way to improving quality of life after SCI.

Statistics Generally the statistical analysis appears sound, but there is some ambiguity that leaves questions of accuracy. For instance, the final paragraph in the Results subsection 'Spinal cord injury causes a transient increase and arrhythmia in plasma CORT' states: “Females with SCI had increased CORT at 7 dpi (vs. pre-surgery; at ZT0; p&lt;0.05); males with SCI showed significant CORT increases at 2 dpi (vs. pre-surgery; at ZT0 and ZT6; p&lt;0.001) and 7 dpi (at ZT0; p&lt;0.05).” Are these post hoc tests (ANOVA) corrected for multiple comparisons, rather than planned t-tests? Since post hoc tests cannot be run after a non-significant ANOVA, is the final statistic listed a planned t-test, or is there a significant main effect as well? Other examples of statistical confusion are listed below under 'Comments to the Author(s)'.

RESPONSE: We thank the reviewer for pointing out this omission. The CORT data did show significant main effects (ANOVA with appropriate post-hoc tests); these details have been added to the text (and to the figure legend):

Page 12: “Female and male SCI rats showed significantly increased CORT levels at acute post-injury times (Females: main effect of injury: F3,62 = 3.606, p &lt; 0.05; males: main effect of injury: F3,70 = 22.584, p &lt; 0.001).”

Comments to the Authors: In this ambitious paper with cross-disciplinary impact, the authors investigate the effects of moderate thoracic contusion spinal cord injury (SCI) in male and female rats on diurnal rhythms of several potentially interrelated physiological, behavioral, and molecular variables. SCI rats showed altered levels and pronounced arrhythmia of these measures at acute and sub-acute time points post-injury. Where tested, approximately normal function was recovered at more chronic time points. The authors conclude that dysregulation of diurnal rhythms may be a key factor contributing to disrupted of quality of life following SCI.

Along with the impressive breadth of the manuscript, the topic of interest is an exciting and underappreciated area in spinal cord injury research and the authors present their findings convincingly. They also note that clock gene expression has not previously been systematically characterized in the spinal cord, an additional novelty. The figures are very clearly presented (colorful, large fonts) and engage the reader without being too distracting.

RESPONSE: We appreciate the positive comments regarding the manuscript.

Several issues dampen my enthusiasm for the manuscript as written, and substantial revisions are encouraged associated with the following points:

1) Assessing clock and inflammatory gene expression at just 2 dpi is a weakness, since other circadian changes persisted through sub-acute time points. The authors state that “The reduced expression of inflammatory markers at 2 dpi may represent an initial remote intraspinal dampening of the immune response, which may precede chronic neuroinflammation that drives neuropathic pain and pathology.” However, it is unclear how long the dampening effect lasts and if normal expression is recovered at sub-acute or chronic time points. If not undertaking additional experiments, the authors are encouraged to defend this particular omission.

RESPONSE: We agree that assessing clock and inflammatory gene expression at 2 dpi alone is a limitation of the study. Reviewer #1 also mentioned that only two timepoints were studied. Unfortunately, all of the authors except the senior authors have relocated since completing these experiments. Thus, undertaking additional experiments would be challenging. In addition, we had limited funding related to this project, which restricted our ability to complete large numbers of groups. We added a paragraph to the Discussion to acknowledge these limitations and suggest the possibility of inclusion in future research.

Page 26-27: “...disruption of circadian outputs persisted beyond 2 dpi, including rhythms of serum CORT, body temperature, and activity (all were disrupted until at least 7 dpi) - yet our study only assessed gene expression changes at 2 d post-surgery. Gene expression was assessed at 2 d post-surgery because robust changes in the circadian system were seen at this time, and because early changes in lesion pathology and inflammation likely have strong effects on recovery trajectory (Noble et al., 2002; Kigerl et al., 2009; Gaudet et al., 2018; McCreedy et al., 2018). It is also important, however, to understand how clock and inflammatory gene patterns shift through sub-acute and chronic times post-surgery. In the future, studying circadian gene expression changes at additional times post-SCI would provide more information about the robustness and duration of SCI's effects on the circadian system.”

1 cont.) It is also unclear how changes in diurnal rhythms correspond to the development of pain since no post-SCI tests of pain sensitivity were performed.

RESPONSE: It is true that neuropathic pain was not assessed, but it is still possible that changes in the lumbar spinal cord are related to later development of neuropathic pain. To help clarify (but not over-state) our point, we added to a sentence in the Discussion:

Page 26: “At later times post-SCI, excess below-level intraspinal inflammation likely contributes to SCI-elicited neuropathic pain (Hains and Waxman, 2006; Detloff et al., 2008), and we found that this contusion model causes neuropathic pain symptoms by 14 dpi in female and male rats (Gaudet et al., 2017).”

1 cont.) Similarly (but less importantly), why was gene expression only assessed at two diurnal times (ZT6 and ZT18)?

RESPONSE: Reviewer #1 also mentioned that only two timepoints over 24 hours after spinal cord injury were collected, and we acknowledge that this is a limitation of the study. As described above, the number of timepoints was limited because of the number of animals required and due to the logistics of performing the SCIs and post-SCI care at manageable intervals. We added text to the Discussion:

Page 26: “Here, two timepoints (middle of the light (ZT6) and dark (ZT18) phase) were used to assess clock and inflammatory gene expression at 2 d post-SCI. This is a limitation of the study, since with two timepoints it is not possible to make more broad conclusions about the effects of SCI on circadian phase, amplitude, and average expression. Future studies could examine expression at additional times of day.”

1 cont.) [Why was] the 42 dpi time point only occasionally measured? This latter chronic time point should be mentioned in the Methods (e.g. under 'Locomotor testing', where time points are not listed).

The 42 d timepoint was occasionally measured because initial experiments showed that many circadian measures had (approximately) normalized by 14 d post-surgery. For example, for the activity and body temperature study, we collected data for ~56 d post-surgery; however, daily rhythms in these measures had recovered by 14 d post-surgery (so data from later times were not shown). For studies completed after this (i.e., the glucocorticoid and gene expression studies), we did not study the 42 d timepoint.

As suggested, we included details about locomotor testing timepoints:

Page 7: “Locomotor recovery was assessed using the Basso-Beattie Bresnahan (BBB) scale (Basso et al., 1995) (female sham n=6, SCI=5; male sham n=7, SCI n=5) by two condition-blind observers prior to surgery; and at 1, 4, 7, 10, 14, 21, 28, 35, and 42 d post-surgery.”

2) Discussion: The authors are encouraged to expand on interrelationships between diurnal rhythms after SCI and perhaps speculate in more detail on underlying mechanisms and prime therapeutic targets, similar to the proposed cross-talk between circadian and inflammatory pathways as a “mechanism” or contributor to observed changes. Generally, the causal relationship between impairment of diurnal rhythms after SCI and overall post-injury recovery could be explored in greater depth, as could the discussion of other outcomes that could be indicative of recovery (e.g. long-term mortality, suboptimal repair, tests of chronic pain or 'wellness'). Although several future directions are proposed, could telemetry or home cage monitoring be used to measure other aspects of physiology as well (heart rate, respiratory rate, blood pressure) to get an autonomic “readout” of direct neural control? And/or is there an easy way to measure the impact on downstream peripheral clocks?

RESPONSE: Several important points are raised here. To address these issues, we added a paragraph to the “Future directions” portion of the Discussion:

Page 27-28: “Our study highlights that SCI disrupts whole-body functions, yet unresolved questions remain about post-SCI circadian disruption and related potential therapies. As shown in Fig. 8, SCI alters regulation of several circadian outputs. Daily rhythms are entrained by light input to the retina influencing the SCN, which is likely protected from changes caused by SCI (e.g., SCN neurons lack CORT receptors (Balsalobre et al., 2000; Woodruff et al., 2016), unlike most cells of the body). However, peripheral rhythms are synchronized and entrained by other circadian oscillators - including CORT, body temperature, activity, and autonomic circuits (Buijs et al., 2016) - that are perturbed by SCI. Therefore, the disruption of circadian outputs could contribute to more widespread circadian disruption throughout the body. Here, a moderate T8 spinal cord contusion was used; future studies could establish whether more severe injuries or injuries at other spinal levels (e.g., cervical or high thoracic, which would more strongly perturb autonomic function (Inskip et al., 2009)) differentially alter post-injury circadian dynamics. In addition, our study did not address whether circadian disruption was simply a result of injury, or whether disruption itself further exacerbated outcomes. Future studies could determine whether amplifying post-SCI rhythms using timed exercise, feeding, light-dark schedule manipulations, injections of circadian-related drugs, etc. could improve recovery measures (discussed more below). Strengthening of post-SCI rhythms could also influence other long-term recovery measures, such as survival, neuroprotection, chronic pain, autonomic function, and general health. Further, since altering circadian rhythms affects other aspects of metabolism, metabolic cage telemetry and calorimetry (Gaudet et al., 2016) could reveal whether boosting post-SCI rhythms improves short- and long-term metabolic health. Finally, to better understand the effect of SCI on circadian rhythms, SCI researchers could adopt other models and strategies frequently used in circadian research - such as the PER::luciferase transgenic rodent, which uses the clock gene promoter from Per to express the bioluminescent luciferase gene and enables studying rhythm synchrony/dysregulation in vitro (Yamazaki et al., 2000) and in vivo (Tahara et al., 2012).”

3) Statistics: The specifics of which statistical tests were used for which data sets and comparisons are sometimes left out, adding confusion and precluding determination of their accuracy (e.g. appropriate correction for multiple comparisons). One example is in the Results subsection 'Spinal cord injury alters daily rhythms in body temperature', where the authors mention that “SCI rats displayed no significant differences vs. sham at 7 dpi or 13 dpi, but did show significant disruptions at 14 dpi... (13-14 dpi: ANOVA, no main effect of treatment: F1,203=1.147, p&gt;0.05; sham v. SCI: 14 dpi: ZT3, ZT6, ZT12, ZT15 - p&lt;0.05).” With no main effect of treatment at 13-14 dpi, what is the basis of subsequent comparisons at 14 dpi (e.g. planned tests)? There are similar issues in a few other sections. Also, sometimes the use of CircWave for rhythmicity tests is clearly indicated, and sometimes it is not. And I assume that the significant results indicated in figures stem from ANOVAs followed by post-hoc tests.

RESPONSE: We appreciate the reviewer highlighting this point of confusion, so that it could be better clarified. In the Methods, we specified the use of post-hoc tests after ANOVAs (rather than the incorrect use of t-tests): “Holm-Sidak post-hoc tests were completed when appropriate for all tests involving more than two groups.” This was also clarified in the appropriate figure legends. In cases where a main effect (by ANOVA) was not found, discussion and mention of significant differences (including related asterisks on the body temperature panels) has been removed as this is inappropriate (as corrected by the reviewer). For instance, male rats' 14 dpi body temperature showed no main effect (ANOVA), so completing and describing the post-hoc would be invalid - we removed asterisks and any inappropriate discussion of these results.

4) What is the authors' rationale for using only lumbar spinal cord (L4-L5) for uninjured spinal cord analysis, vs. thoracic and lumbar for injured cord analysis (with injury epicenter being thoracic)? This concern is mitigated by the inclusion of sham rat thoracic spinal cords. Nevertheless, this inclusion could have been valuable given the general lack of understanding of rhythmic gene expression in the spinal cord that the authors point out. Also, re: “L4-L5 spinal cord was chosen as a distal spinal cord site, because it integrates hindpaw nociceptive information.” This information may be better situated early in the manuscript (i.e. Methods).

RESPONSE: We agree that the variation in clock and inflammatory gene expression between spinal cord sites could be interesting, but is beyond the scope of this study. The changes elicited by SCI were likely to be stronger than the minor differences likely to be seen along the neuraxis. We added more description to clarify the important points raised above in the Methods:

Page 9: “Tissues were flash frozen for PCR (L4-L5 was used for uninjured spinal cord analyses; T8 (lesion) and L4-L5 were used for post-surgery analyses). L4-L5 was used as a distal spinal cord site because it integrates hindpaw nociceptive information, which could provide useful information for future studies. L4-L5 was used for the uninjured analyses, since the T8 injury site was expected to have major shifts in gene expression (SCI vs. sham, given that the lesion was present), whereas the lumbar spinal cord was predicted to have more minor SCI-elicited changes.”

5) There are minor textual errors (grammar, terminology, repetition) throughout the manuscript. Please proofread more thoroughly and correct or clarify. As one example, several of the figures include both sham and SCI rats under headings such as “dpi” (days post-injury) and “post-SCI” (e.g. Figures 6B,C and 7B,C). In the Significance Statement, the two consecutive sentencing starting “Moderate SCI in rats caused...” and “Our data reveal that SCI perturbed...” seem to basically repeat the same idea. The Figure 3 legend has a similar issue, defining both “~” and “Black ~” (it appears all symbols are black in this figure). Relatedly, the last sentence in the Introduction says “These results suggest that failing to optimize diurnal rhythms after SCI could exacerbate tissue damage and neuroimmune dysfunction.” I do not believe the authors address how changing diurnal rhythms could exacerbate tissue damage.

RESPONSE: We thank the reviewer for highlighting these minor errors. These have now been corrected. We changed “dpi” to “d post-surgery” where appropriate - both in the manuscript itself and in the figures. We also removed the final sentence of the Introduction, and deleted the repetitive sentence in the Significance Statement.

6) In the Abstract the injury is described as “moderate-to-severe contusion SCI” whereas in other sections it is specifically called “moderate.” Please correct and also state in Methods whether the injury was midline or lateralized. Also, it appears analgesics were withheld following surgery to prevent potential confounds associated with pain following SCI. If this is incorrect, please update the corresponding methods subsection (“Surgery and animal care”).

RESPONSE: We thank the reviewer for pointing out the discrepancy with the SCI severity. We changed the description in the Abstract to “moderate SCI,” and included in the Methods that it was a midline injury.

As mentioned, analgesics were withheld, and this is now addressed:

Page 6: “Initial related studies involved pain testing, and inflammation was also studied, so analgesics were not given to any rats for consistency and limiting confounds.”

7) Individually housed rats were used for radiotelemetric measures (body temperature and locomotor activity), while rats were pair-housed for collection and measurement of plasma corticosterone. While this was likely a practical necessity stemming from recording procedures, given that individual housing is a recognized stressor this limitation should be recognized and perhaps briefly addressed in the Discussion. Similarly, differences in design between experiments using male and female subjects are honestly addressed by the authors and are a minor weakness that could contribute to differences observed between sexes.

RESPONSE: We agree that the difference between pair-housed rats (as used in most studies here) and individually-housed rats (used in the telemetry studies) could be a limitation. As suggested, we added a brief statement to the Discussion.

Page 24: “Another distinction between studies described herein is that the telemetry study was completed with individually-housed rats (to enable measuring a single rat's temperature and activity), whereas all other studies were completed with rats housed in pairs. This is important to note, given that housing rats individually is a stressor (Von Frijtag et al., 2000; Westenbroek et al., 2005) and can impair recovery after CNS injury (Craft et al., 2005; Karelina et al., 2009; Gaudier-Diaz et al., 2017). Furthermore, single housing may reduce relative activity (vs. having a cagemate) and therefore likely worsens other aspects of recovery (e.g., locomotor recovery) (Van Meeteren et al., 2003).”

Discussion of differences in treatment of the telemetry experiments is important, and was also mentioned by Reviewer #1. We clarified further in the Results:

Page 12: “(The studies on male and female rats were completed consecutively, due to limited number of telemetry receivers - see Materials and Methods for details.)”

In addition, we added a paragraph to the Discussion:

Page 23-24: “It is important to note that we had limited telemetry receivers, so we completed the body temperature and activity transmitter study separately in males and females. In addition, limitations were noted after completing the male study and methods were improved for the female portion, which could have affected the consistency of the results. Despite this, the results were remarkably similar between males and females - both for sham and SCI rats - and differences existed between sham and SCI groups. Our experience and data also highlight that typical post-surgery rodent care and behavioral testing in many research labs (e.g., twice-daily rodent care during the light/inactive phase) likely further disrupts daily rhythms (as well as sleep), and could be a confound to the factor under study (e.g., (McEwen, 2006). Future SCI studies could improve design by having the daily injections and major care immediately before the active phase, and by having a second potential daily care time during the dark or at another minimally disruptive time (using shifted light schedules in animal housing, if helpful). In addition, given that intense rodent care during the beginning of the inactive phase is likely a strong disruptor of rhythms, future studies could compare whether this type of disruption affects anatomical and neurologic outcomes compared to a ”circadian-optimized“ care schedule. These findings would have translational relevance, since early post-SCI patients in hospitals likely experience excess circadian disruptions (that could be improved with relatively simple interventions).”

8) Link to software (CircWave) does not appear to work when entered into an internet browser. The first link I do find upon searching for CircWave states that “CircWave analyses independent circadian data (ie, one individual contributes one data point). Typically such data sets are produced in circadian brain research where techniques such as micro array chips, in situ hybridisations, immunocytochemistry, northern, western, and soutern blots, etc...are used.” I do not believe that was the case here, i.e. no repeated data points in a given animal. An updated link would remedy confusion over how cosinor analysis was performed.

RESPONSE: We also addressed this in the first answer above.

We have updated the link to the software. In further considering effective use of the software, we agree that it is inappropriate to use the program for repeated data points. As such, we have removed inappropriate analyses and related discussion of rhythmicity (e.g., for glucocorticoids, temperature, and activity figures). CircWave is appropriate for analyzing the gene expression data presented in Fig. 6 and 7. We updated the description of circadian analysis using CircWave to be more specific:

Page 10: “Circadian data were analyzed using CircWave (https://www.euclock.org/results/item/circ-wave.html), which assesses independent circadian data (i.e., one individual contributes to a single data point) to identify waveforms and acrophases (rhythm peaks).”

Furthermore, to better understand how SCI altered circadian rhythms, we performed additional analysis on the post-SCI temperature data and have added a new figure (Figure 4). The data show that males, in particular, have SCI-elicited disruptions in temperature rhythms: SCI males (but not females) show significantly advanced acrophases of temperature rhythms. Discussion of this is included in the Results:

Page 14-15: “To understand more broadly whether SCI influenced temperature acrophase, daily temperature was processed in ClockLab in grouped multi-day ”bins“ (Fig. 4). Prior to surgery, peak daily temperature phase occurred in females at an angle of 263.5o (or ZT17.8) and in males at 275.2o (or ZT18.3). After surgery, females with SCI showed no significant difference in phase from shams (p &gt; 0.05; two-way RM ANOVA with Holm-Sidak post-hoc). In contrast, males with SCI displayed significantly advanced temperature phases: SCI males had earlier temperature acrophase starting at 2-5 d post-surgery (ANOVA main effect of treatment: F1,81=43.299, p &lt; 0.001) (2-5 d post-surgery: male-sham, phase angle 261.9o {plus minus} 3.4o (=ZT17.5); male-SCI, 208.3o {plus minus} 15.3o (=ZT13.9), p &lt; 0.05) and these SCI-advanced rhythms continued through all 5 d bins examined through 26-30 d post-surgery (26-30 d post-surgery: male-sham, 268.7o {plus minus} 4.9o (=ZT17.9); male-SCI, 249.9o {plus minus} 4.1o (=ZT16.7), p &lt; 0.05). At 31-35 d post-surgery and beyond, male SCI rat temperature acrophases were not significantly different from male sham rats (e.g., 31-35 d post-surgery: male-sham, 269.5o {plus minus} 3.5o (=ZT18.0); male-SCI, 257.2o {plus minus} 4.o (=ZT17.1), p &gt; 0.05).”
